# Recent advances and potential applications for metal-organic framework (MOFs) and MOFs-derived materials: Characterizations and antimicrobial activities

**DOI:** 10.1016/j.btre.2024.e00837

**Published:** 2024-03-20

**Authors:** Muhammad Hubab, Mohammad A. Al-Ghouti

**Affiliations:** Environmental Science Program, Department of Biological and Environmental Sciences, College of Arts and Sciences, Qatar University, State of Qatar, Doha, P.O. Box: 2713, Qatar

**Keywords:** Metal-organic frameworks (MOFs), Antimicrobial activities, Antifungal, Antibacterial, Antiviral

## Abstract

Microbial infections, particularly those caused by antibiotic-resistant pathogens, pose a critical global health threat. Metal-Organic Frameworks (MOFs), porous crystalline structures built from metal ions and organic linkers, initially developed for gas adsorption, have emerged as promising alternatives to traditional antibiotics. This review, covering research up to 2023, explores the potential of MOFs and MOF-based materials as broad-spectrum antimicrobial agents against bacteria, viruses, fungi, and even parasites. It delves into the historical context of antimicrobial agents, recent advancements in MOF research, and the diverse synthesis techniques employed for their production. Furthermore, the review comprehensively analyzes the mechanisms of action by which MOFs combat various microbial threats. By highlighting the vast potential of MOFs, their diverse synthesis methods, and their effectiveness against various pathogens, this study underscores their potential as a novel solution to the growing challenge of antibiotic resistance.

## Highlights

• Microbial infections are a serious global health problem.
• MOFs are promising new antimicrobial agents.
• MOFs have been shown to be effective against bacteria, viruses, and fungi.
• MOFs have the potential to address the growing challenge of antibiotic resistance.

## Graphical abstract

**Figure:**
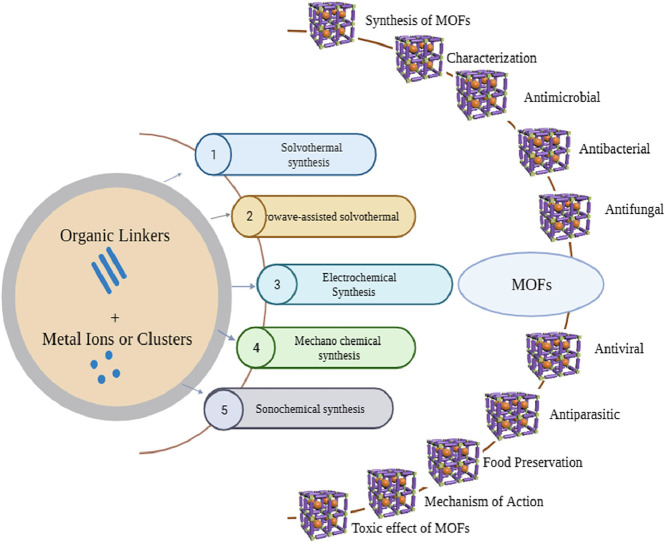

## 1 Introduction

The rising antibiotic resistance of microbial pathogens has become a major issue. There are several reasons for antibiotic resistance including misuse and overuse of antibiotics, variation in the metabolic pathway or targeted site of an antibiotic, decrease in the drug accumulation in cells, or its inactivation. New approaches are required for this reason and there should be an urgent development of active antimicrobial materials Dizaj et al. [1]. Different initial infections caused by different bacteria are limited to a local area in the initial stages however several communicable diseases or pathogens transfer from one person to another person through different sources such as air, water, physical contact, body fluid with other infected objects, etc. Qureshi et al. [2].

**Fig. 1:**
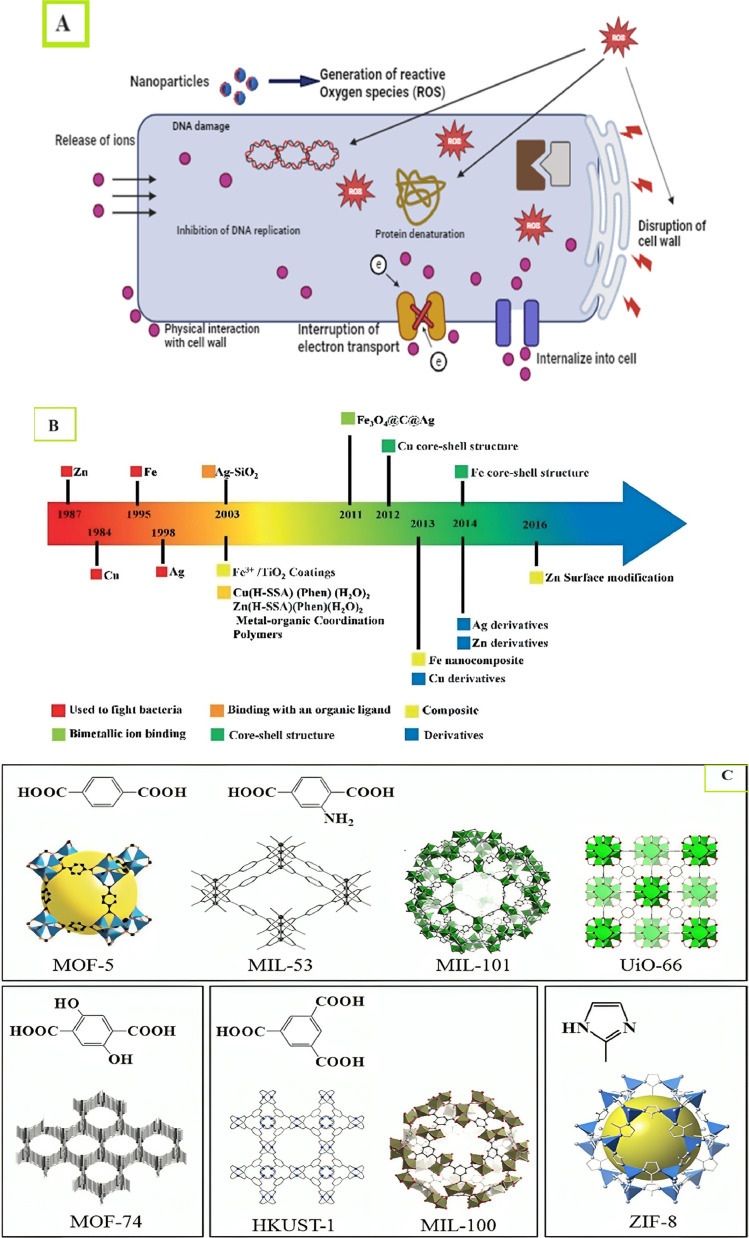
(A) Demonstrates the several suggested antibacterial mechanisms of the Nanometals, (B) Advancements in the creation of materials based on MOFs for antimicrobial therapeutic systems [9], (C) exemplary MOF structures [10].

The bacteria, which are resistant to different antibiotics, cause severe infections in humans, which is a big challenge around the globe [3,4]. Alexander Fleming discovered penicillin in 1928, which was a successful event for researchers. However, due to misuse and overuse of antibiotics over time, different bacteria started to show resistance to the discovered antibiotics [5,6]. Metal-organic frameworks (MOFs) especially contain nickel oxide (NiO), zinc oxide (ZnO), cobalt oxide (CoO), copper oxide (CuO), and copper(I) oxide (Cu_2_O) in their nano-forms, which show the best results against different microorganisms and can be considered as biocide agents [7,8]. Dizaj et al. [1] demonstrated several suggested antibacterial mechanisms of the nanometals (Fig. 1A).

Wongpreecha et al. [11] conducted a study where chitosan-based MOFs were used against *Staph. Aureus* and *Escherichia coli* as antibacterial agents. Ultimately, they failed to find any zone of inhibition and concluded that it was ineffective; this might be due to a variety of microbial, chitosan (CS), or environmental variables Zhang et al. [12] conducted the same investigation, using chitosan to create silver-metal organic frameworks (Ag-MOFs@CS and Ag-MOF). Fig. 1B shows the advancements in the creation of materials based on MOFs for antimicrobial therapeutic systems [9]. Fig. 1C (C) shows exemplary MOF structures [10].

Cu nanoparticles attracted the attention of scientists due to their unique physical, chemical, and biological properties and low preparation cost [[13], [14], [15]]. Cu nanoparticles were used against *Shigella strains, Klebsiella pneumoniae, Salmonella paratyphi* and *P. aeruginosa*. The Cu nanoparticles showed potential activities against these microbes by crossing the cell membrane and damaging the vital bacterial enzyme, which lead to the critical death of the cell [16]. Azam et al. [17] studied the antibacterial activity of copper depending on size. The study was completed against both gram-positive bacteria including (*Bacillus and S.aureus*) *and* gram-negative (*E. coli* and *Pseudomonas auroginosa*). The results showed the inhibitory effects against both groups. Their conclusion revealed that the bacteriocidal activity of Cu nanoparticles depends on the particle size and concentration of the copper particles in the growth medium of the bacteria. Bacterial growth can be inhibited by passing the nanoparticles through the pores present on the cell membrane of bacteria [17]. There are several other mechanisms. Fig. 1A shows an antibacterial mechanism of copper nanoparticles.

MOFs are suitable for use as antibacterial properties due to their unique properties. MOFs release and control the antibacterial agents by various methods such as the ligand releases from the MOFs [18]. Several metal ions released from the MOFs such as Cu, Zn, and Ag [19] active species released against the bacteria and entrapped within the MOFs [20] or some antibacterial agents released after the modification in the MOFs [21]. The development in MOFs against bacteria is mentioned in Fig. 1B.

MOFs represent various attractive structures, which can be prepared by various methods. Yagi et al. [22] reported MOF-5 in 1995 and attracted huge attention due to its porous structure [23]. When it was revealed that MOF-5 has weak hydrothermal stability, HKUST-1 attracted huge attention [24] due to its excellent stable condition and resistance to moisture, easy synthesis, and outstanding thermal stability. MIL-101 is adopted most for adsorption and catalysis. It is a robust MOF and has a large surface area. It can be prepared typically by hydrothermal method by using chromium salt and H_2_BDC in an autoclave [25].

MOF-74 is attracted due to its high adsorption capacity of CO_2_ under atmospheric pressure conditions [26]. MIL-53 is attracted due to pore expansion and behavior of contraction and interaction with guest molecules [[27], [28], [29]] in separation and storage of gas whereas MIL-100 is interested because it has high stability [30] and UiO-66 is attracted due to its hydrothermal stability [28]. Various exemplary structures of MOFs are given in Fig. 1C.

In the modern world, bacteria started to show resistance to different antibiotics whether the bacteria are gram-positive or gram-negative. Both organisms use the same strategy to evade host cells, immunity, and antibiotics. Gram-positive bacteria are easy to treat as compared to gram-negative because a thin peptidoglycan surrounds the gram-negative bacteria further enclosed by an outer membrane, which prevents antibiotics from entering the bacterial cell [31].

**Fig. 2:**
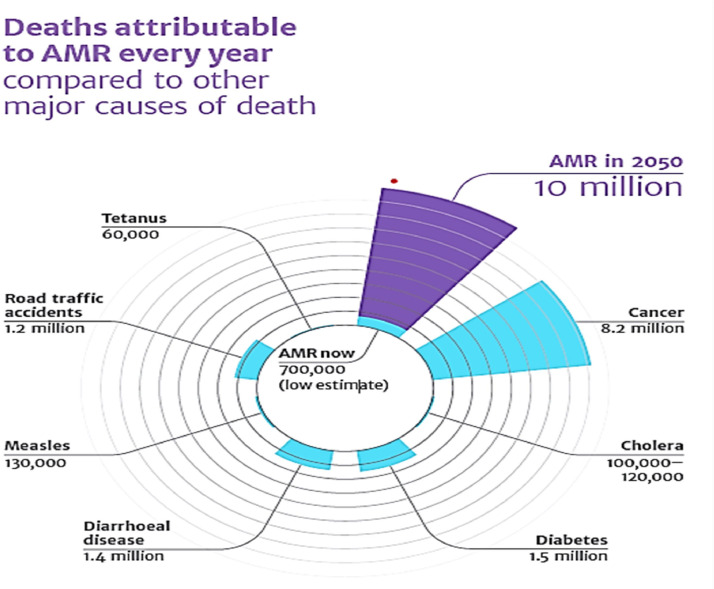
Predictions about death due to AMR for the year 2050 2050 [32,33].

There are many drugs, antibiotics and other antimicrobials developed and discovered to use them against pathogens, the misuse, overuse, and self-medications of these antibiotics led to antimicrobial resistance (AMR), leading to bacterial growth with multidrug resistance (MDR). This situation poses an alarming threat to the entire world. Recently, the World Health Organization (WHO) surveyed that more than 700,000 individuals (about half the population of Hawaii) are threatened by bacterial infection due to antibiotic resistance, and it is expected that the number will rise to 10 million by the year 2050 [32,33]. The same prediction about the death rate is mentioned in Fig. 2.

AMR increases mortality and morbidity, the financial burden on the healthcare system, causes major losses in the economy and is predicted to become the major cause of death by the year 2050. Shown in the figure [[34], [35], [36]].

According to recent research by Liu et al. [9], a wide range of antibacterial MOFs is prepared and used widely due to their best results against different microorganisms. These MOFs are connected to various material bases. As an antibacterial agent, MOFs have a broad spectrum of action that is long-lasting and effective against both gram-positive and gram-negative bacteria. Combating various disease-causing microorganisms is seen as beneficial and acceptable in biomedical research and associated fields [37].

**Fig. 3:**
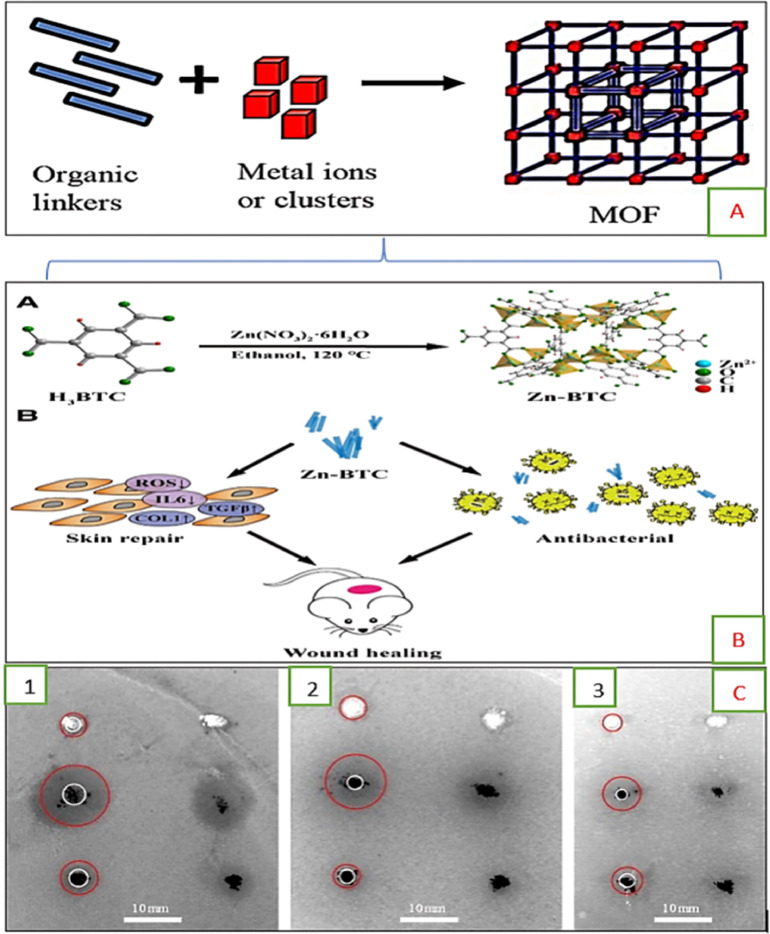
(A) Basic structure of metal-organic framework (MOF), (B) Zn-BTC fabrication and its probable anti-inflammatory and antibacterial capacity, further lead to promote wound and skin healing [40], (C) disc diffusion methods was performed to find out antibacterial activity.

MOFs present a basic structure. MOFs in a way that a class of coordination polymers is represented by MOFs, which consist of inorganic ligand hybrid frameworks, that present great rate permeability. MOF structure is composed of linkers such as organic ligands and connectors such as metal centers as shown in Fig. 3(A) [38,39].

Because of their remarkable performance and various structural alterations with distinct purposes, MOFs have recently been classified as third-generation antimicrobial agents [41]. The best antibacterial activity was demonstrated by other investigations conducted by other researchers on various MOFs-based materials, such as zinc basis on MOFs (Zn/Al-terephthalate MOF) [42,43]. In addition to all of this, [40] created zinc-based MOFs (Zn-BTC) as illustrated in Fig. 3(B) by heating trimesic acid (H_3_BTC) and adding Zn (NO_3_)_2_. These were then utilized in-vivo and *in vitro* to verify the compounds' biocompatibility, antioxidant, wound-healing, anti-inflammatory, and antibacterial qualities. This study further shows that (Zn-BTC) Zn-based MOFs were prepared in white powder form to improve the healing of skin wounds. Zn-BTC was used against *E. coli* and *Methicilne-resistant staph. Aureus* (MRSA). The method of co-culture was used to examine the ability of Zn-BTC against bacteria. The growth of targeted organisms was inhibited by the Zn-BTC, and the number of bacteria was decreased. MRSA was used to infect the wounds of rats. After the wound area observation at 0, 3, 7, and 14 days (about 2 weeks), it was noticed that the wound healing effect was expressed and improved by Zn-BTC.

The most important and recent research was done by [44] by using cobalt-based MOFs against some different microorganisms including *P. Putida, S. aureus,* and *E. Coli.* Their results demonstrate that cobalt-based MOFs can be used as an antibacterial agent. Using the disc diffusion technique, the antibacterial activity of three distinct MOFs was assessed. Because cobalt ions are released in an antibacterial solution in a regulated manner, as seen in Fig. 3(C), MOFs based on cobalt ions demonstrated the greatest results. Whereas silver coordination polymer (AgTAZ) provided the weakest results for bacterial growth inhibition. Results of the initial experiments showed that both cobalt-based imidazole (Co-SIM-1) and zeolitic imidazolate framework (ZIF-67) easily diffused in the used medium and inhibited the growth of all three bacterial strains. Besides all this, their preparation was simple, with easily available materials and cost analysis. This can become an affordable antimicrobial agent in the future.

The following bacterial strains were used (1) S. cerevisiae, (2) P. putida, and (3) E. coli with (silver coordination polymer) AgTAZ, (zeolitic imidazolate framework) ZIF-67 and (cobalt-based imidazole) Co-SIM-1. The white circles point out the material, which is deposited while the red circles are the confirmation of the zone of inhibition, which shows the action of cobalt-based MOFs against the bacteria Aguado et al. [44].

Shams et al. [45] investigated the antibacterial properties of Cu/benzene-1,3,5- tricarboxylic acid MOF composed of copper and trimesate ions against *staph. Aureus* and *E. coli*. It showed the possibility of Cu/H_3_BTC MOFs to be used against different bacteria including *E. coli* and *S. aureus*. Cu/H_3_BTC MOFs target their DNA and lead the cell to death by disrupting the cell membrane. Copper is always known as a trace element necessary for maintaining bioactivity. This is always used as a long-lasting antimicrobial agent in the early ages. It is considered the strongest and most popular copper-based MOF material [46].

MOFs are highly significant in today's world and have a wide range of uses, including food packaging, the environment, health, and the absorption of dangerous chemicals from liquids. Table 1 provides an overview of their uses. The current review, however, brings attention to the historical antimicrobial agents and latest developments in the field of MOFs and comprehensive relevant literature available from 1986 to 2023. This is to provide a complete overview of the vast potential inherent in MOFs and MOFs base materials, their methods of synthesis, characterization, and antimicrobial activities against different microbial agents including bacteria, viruses, fungi, and parasites as well as mechanisms of action. All the potential mechanisms and production of antimicrobial agents are identified and explained. Lastly, the current review highly recommends that MOFs and materials based on MOFs mostly be possible solutions for the current challenges of antibiotic resistance.

**Table 1:** Current and other different studies about MOFs and their applications.

S. No.	Title	Scope	References
1	Synthesis of magnetic metal-organic framework (MOF) for efficient removal of organic dyes from water	The study focuses on an excellent magnetic response of MOFs by removing organic substances from water	[47]
2	Removal of hazardous organics from water using metal-organic frameworks (MOFs): Plausible mechanisms for selective adsorptions	This study aims to purify contaminated water through adsorption by removing different harmful substances such as organic substances. As well as plausible interaction or selective adsorption and mechanisms of adsorption are briefly summarized.	[48]
3	Multifunctional metal-organic frameworks: from academia to industrial applications	The study reveals how MOFs are used in laboratories and what their role is in removing hazardous gases from the environment.	[49]
4	Applications of metal-organic frameworks featuring multi-functional sites	The author aims to discuss the multifunctional sites of MOFs such as applications of MOFs in optics, protein production, and CO2 adsorption and synthesis of MOFs.	[50]
5	Emerging Applications of Metal−Organic Frameworks and Covalent Organic Frameworks	This study shows the elevated surface area of the MOFs, storage, adsorption, and sensing and detection capacity.	[51]
6	Applications of water-stable metal-organic frameworks	In this study, MOFs are presented with five major applications conducted in water such as catalysis, adsorption, sensing, membrane separation, and proton conduction.	[52]
7	Metal-organic frameworks: mechanisms of antibacterial action and potential applications	The scope of this article is the activity against the bacteria exhibited by MOFs as well as MOFs acting as a carrier of antibacterial substances.	[37]
8	Application of metal−organic frameworks	This article includes the MOF's synthesis and various applications in Biomedical sciences, gas delivery and storage, catalytic, and sensing applications as well as water and air purification applications	[53]
9	Synthesis and applications of MOF-derived porous nanostructures	This study includes the most current development/progress of MOFs as precursors for several nanostructure preparations their possible future applications devices and their process related to energy	[54]
10	Recent advances in the construction and analytical applications of metal-organic frameworks-based nanozymes	This study plans to explain the most current development of MOFs-based nanozymes construction and their prime applications in chemical sensing and bio sensing.	[55]
11	The Applications of Metal−Organic Frameworks in Electrochemical Sensors	In this study, the author presents the recent progress report about the fresh performance of electrochemical sensors and general sensing principles which is achieved by research in MOFs.	[56]
12	Green applications of metal-organic frameworks	This article highlights several applications of green chemistry including catalysis, storage, and solar energy conversion as well as air and water pollution remediation.	[57]
13	Improving MOF stability: approaches and applications	This article summarizes the recent synthesis and design of the MOFs and MOFs-based materials along with the stability and application of MOFs including separation, adsorption, Fluorescence sensing, and Heterogeneous catalysis, etc.	[58]
14	Applications of Metal-Organic-Framework-Derived Carbon Materials	The authors explain the applications of MOF-derived carbon materials and applications in batteries including sodium-ion batteries (SIBs), lithium-ion batteries (LIBs), lithium-sulfur batteries (LSB), oxygen evolution reactions (OER), hydrogen evolution reactions (HER) and reactions including oxygen reduction reactions (ORR) etc	[59]
15	Synthesis of metal-organic frameworks (MOFs) and its application in food packaging: A critical review	This study aims at the food shelf life through the MOFs incorporation, water absorption, and O2 molecules from food packaging.	[60]
16	Application of MOF-based materials in electrochemical sensing	The article demonstrates the most current developments in MOFs and MOFs-derived materials regarding their application for electrochemical sensing.	[61]
17	Recent advances and potential applications for metal-organic framework (MOFs) and MOFs-derived materials: characterizations and antimicrobial activities	The purpose of this study is to find out about the synthesis of MOFs and MOFs based materials with different methods, their characterizations, and different applications regarding antimicrobials including different MOFs and MOFs based materials that act as antibacterial, antiviral, antifungal, and anti-parasitic agents and helps in food preservations.	Current study

MOFs show considerable potential for transporting therapeutic agents to the infected sites. Taking advantage of their versatile characteristic, the controlled release of antimicrobial agents is facilitated by MOFs, ensuring sustained and consistent activity and reducing the risk of resistance development. Recently, the MOF application, especially nano MOFs, was developed in biomedical. MOFs possess unique features and are considered as promising agents for drug delivery for cancer therapy. MOFs overcome some of the challenges for cargo delivery drugs due to some unique features such as a diverse range of metal ions/clusters and structure of organic linkers that provide MOFs with different morphologies, changed compositions, distinct chemical features, and adjustable sizes. These characteristics make MOFs suitable to accommodate a broad spectrum of drugs with different physiochemical properties. Recently Hf (IV)-based MOFs were introduced in 2012 and have gained significant attention among materials and biomaterials chemists due to their diverse applications. Particularly, Hf (IV)-MOFs containing Z-Hf metal content present new options in cancer treatment [62].

Furthermore, MOFs possess large pore size and high surface area making them suitable for accommodating high capacity of molecules and biomolecules including nucleic acids and enzymes. MOFs release drugs in a controlled way to ensure them for safe delivery of drugs. The coordination bond in MOFs contributes to good biodegradation [63]. The rapid advancement of bio-imaging technologies serves as a crucial tool to investigate the metabolic functions and pathological characteristics of biological tissues. This progress promotes the diagnosis of diseases. Imaging agents including small fluorescent molecules and contrast agents are used to produce signals and strengthen signal contrast to targeted tissues. MOFs-based nanocomposites are used on a large scale in computed tomography (CT), Positron emission imaging (PET), fluorescence imaging (FL), and magnetic resonance imaging (MRI) [64].

The use of MOFs in enzymes is another biomedical application. Natural enzymes are biological macromolecules produced by living tissues but there are some drawbacks of enzymes such as easy deactivation, high cost, less tolerance to some metal ions and some solvents, and very limited application in industrial catalysis. Recently, MOFs have provided efficient support in the immobilization of enzymes. MOFs possess remarkable properties such as porosity, large surface area, excellent chemical and thermal stability, adjustable affinities, and high loading capacities. These features make MOFs suitable for the stability of immobilized enzymes in different applications [65,66]. As discussed earlier, due to the outstanding features of MOFs including their nanometer-scale size, biocompatibility and biodegradability, and extensive surface area, MOFs exhibit significant promise in biomedical applications, including biocatalysis, drug delivery, bioimaging, and biosensing [67].

MOFs have undergone comprehensive exploration in different applications, mostly in biological fields due to their exceptional properties as hybrid composite systems. MOFs have been described as materials consisting of organic and inorganic hybrids, porous coordination networks, and metal-organic and coordination polymers. In the research of nanoporous material, MOFs have stood out as highly attractive substances. With their exceptional combination of extensive surface areas, high porosity, diverse topologies, absence of inaccessible bulk volume, pore sizes, and potential structure capability positions, MOFs as smart alternatives to traditional nanoporous materials in several industrial and scientific domains. The interest in this field strengthened with the MOF synthesis through the reticular design concept in late 1999, despite their initial discovery dating back to 1965 [68]. The values of surface area for typical MOFs range from 1000 to 10,000 m^2^/g. The large surface area of MOFs contributes to the removal of pollution and dyes Li et al. [69] performed the 1st permanent microscopy of MOFs where the Langmuir surface area of the material was described as 310 m^2^/g. Different MOFs with their different surface area and pollutant removal rates are given in Table 2.

**Table 2:** Surface area and pollutant adsorption capacity of MOFs.

**MOFs (Adsorbent)**	**BET surface area (m2 /g)**	**Removal rate**	**Pollutant**	**References**
Gd-PTA	15.14	206.13 mg/g	PO_4_^3−^(Phosphate	[70]
Fe-MOF	128.3	70.02 mg/g	Arsenate	[71]
Ce-UiO-66	1101	793.7 mg/g	Congo Red (CR)	[72]
ZIF-8 (Zn)	978	43.1 mg/g	Methylene Blue (MB)	[73]
Co-MOF	2.23	147.99 mg/g	Methylene Blue (MB)	[74]
CuMIL-101(Fe)	15.48	497.3 mg/g	Naproxen Ibuprofen	[75]
DONA-MOF	15.19	120 637.5 mg/g 3	Gold (III) Au (III)	[40]
		191.27 mg/g	Palladium (III) Pd (II)	
MOF-808	1610	939 mg/g	Perfluorooctanesulfonic acid PFOS	[76]
UiO-66-NH2	919	338.98 mg/g	Hexavalent chromium Cr (VI)	[62]

Great efforts have been made to enhance the chemical stability of the MOFs, particularly focusing on the pH and its stability related to chemical stability. The widespread pH instability leads to the limited practical, commercial, and biomedical applications of MOFs. The scientific community should give greater attention to the pH stability of MOFs [77]. The pH stability of MOFs is defined by their capacity to maintain structural integrity and enduring porosity even following exposure to alkaline and acidic substances. Generally, MOFs with strong coordination bonds can resist and protect their crystallinity from degradation. The breakage of metal-ligand bonds results in the degradation of MOFs. Strategic adoption in metal node selection and the design of organic linkers have been implemented to advance the development of MOFs with enhanced pH stability making them resistant and enabling them to survive in harsh environments [78]. There are a few application areas that have been deployed for pH-stable MOFs. The development of MOFs with pH stability is of great interest for storage applications and gas separations, especially in different basic and acidic impurities in different mixtures of feed gases [79].

With a concentration on “green chemistry”, diverse heterogeneous catalytic reactions are increasingly conducted in aqueous or acidic-basic media. Photocatalytic reactions typically favor an alkaline environment for the efficient generation of labile electrons. Electrochemical reactions, achieved in both basic and acidic mediums, highlight the need for pH-stable MOFs. Biological processes greatly depend on the pH conditions of the system. MOFs designed for biomedical applications must exhibit robust resistance in physiological environments, such as resisting hydrolysis in intestinal alkalinity and stomach acidity over a specific duration. Therefore, the use of pH-stable MOFs in biomedical applications is essential.

On the consideration of material stability, an examination of the operating environment is required including pH substantial efforts have been made to clarify the MOFs' stability in both basic and acidic conditions [80,81]. When the environment is acidic, the degradation of MOFs primarily arises from the competition between metal ions and protons for coordination with organic ligands. In an alkaline environment, the predominant factor contributing to the decomposition of MOFs is the substitution of organic ligands for hydroxide ions. MOFs containing high valent metal ions and carboxylate ligands exhibit stability in an acidic environment but show reduced resistance in the presence of bases. Whereas the MOFs based on soft divalent metal ions and azolate ligands demonstrate greater stability in basics but are resilient in acidic conditions [82].

To advance sustainable development globally, the principles of green chemistry are increasingly being considered in pharmaceutical and chemical production. The focus is to minimize the use of unfriendly environmental energy and materials in the production of MOFs. Various factors are involved in the synthesis of MOFs such as heating techniques and chemicals. Some of the widely used materials are not considered as entirely green; their careful use in MOF production on a large scale is considered important. These challenges are critical for sustainable and eco-friendly production of MOFs on a large scale [83,84].

The green synthesis of MOFs is a convincing research area for sustainable materials. MOFs possess porosity and large surface area with a variety of applications such as drug delivery, catalysis, storage, etc. The green solvent synthesis method utilizes nonhazardous solvents like methanol and ethanol and offers an environmentally friendly approach to the synthesis of MOFs. The different methods of eco-friendly MOF synthesis offer opportunities to customize material for specific applications, supporting the worldwide efforts for sustainable development [85]. Based on the above discussion, MOFs are almost considered eco-friendly. The example given here is about nicotine. Nicotine is released into the environment through various means, either directly or as a byproduct of industrial processes related to the widespread use of nicotine-based products. Its potential toxicity and long-term effect on human health have made it a serious issue around the globe.

Currently, a magnetic sulfur-doped metal–organic framework-235 (sulfur-doped MOF-235@g-C_3_N_4_) has been created through solvothermal synthesis methods using iron terephthalate. This material has been used as a photocatalyst for nicotine removal [86,87] conducted a study where ZIF-8 and its composite ZIF-8@ZnAl-LDH are used for the removal of water pollutants such as dyes. According to their study, the synthesized MOFs demonstrated effectiveness in absorbing and removing dyes such as Malachite green and Methyl Orange from water. Their findings not only presented accessibility to synthesize the MOFs ZIF-8@ZnAl-LDH nanocomposite with customized structure, porosity, and high stability but also extended applications for water pollution treatment.

As discussed earlier, MOFs are versatile materials used in various applications like drug delivery and gas storage. Researchers are exploring ways to make MOF synthesis more eco-friendly by incorporating principles of green biomaterials. Green biomaterials combine existing knowledge from technology, biology, and chemistry to develop sustainable solutions that benefit society. This approach promotes environmental awareness, particularly concerning public health and related technologies. By integrating core principles from green chemistry and biomaterials, researchers are shaping a new era of sustainable material development across various fields like medicine, engineering, and physics.

Green biomaterials are built on principles like minimizing waste, using energy-efficient methods, and employing natural, renewable resources throughout the production process. This includes using eco-friendly solvents and incorporating nature-inspired approaches. Additionally, they emphasize designing materials that are safe, biodegradable, and compatible with the body, while minimizing environmental impact through life-cycle assessments. These principles directly benefit MOF synthesis. By promoting green solvents, waste-free reactions, and the creation of biodegradable and non-toxic MOFs, green biomaterials ensure a more eco-friendly and sustainable approach to MOF production.

## 2 Synthesis of MOFs

**Fig. 4:**
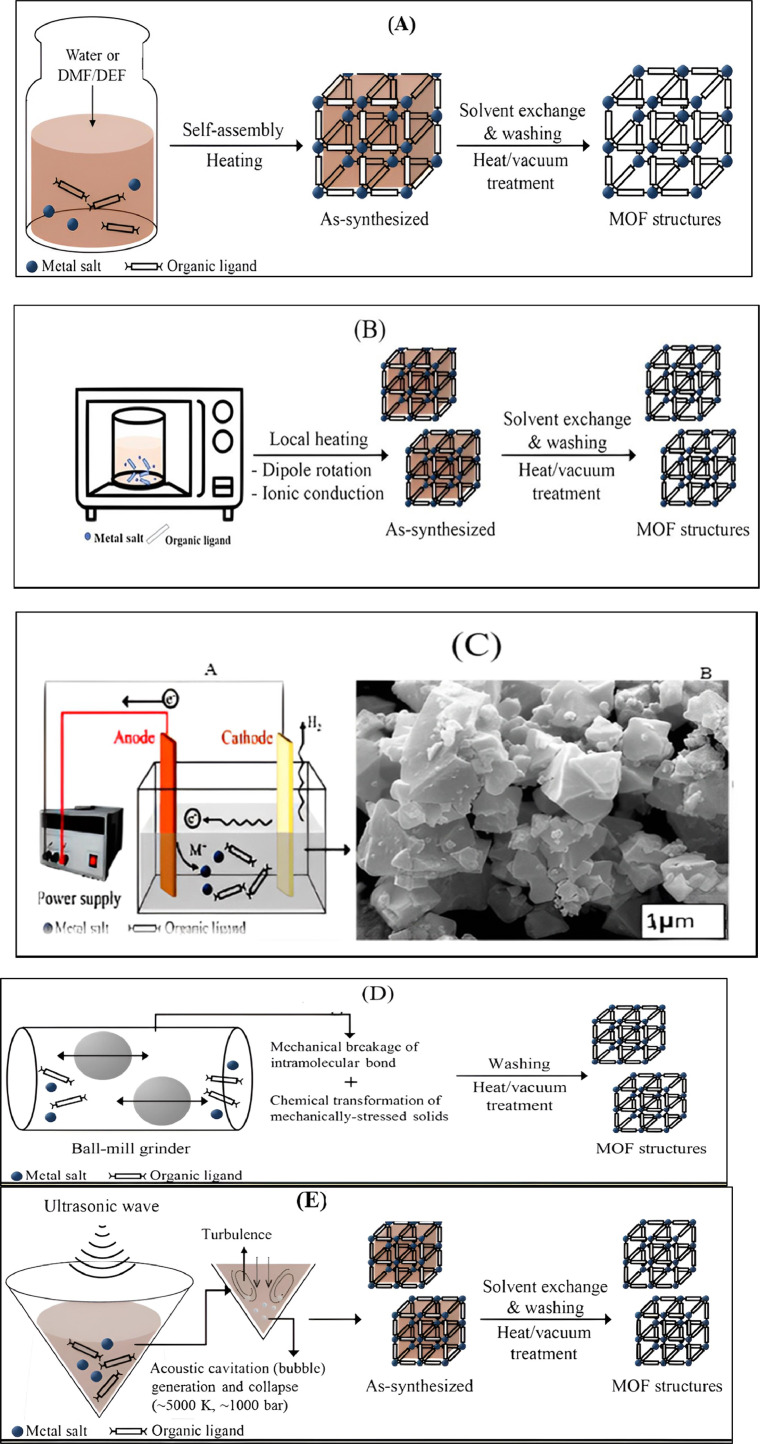

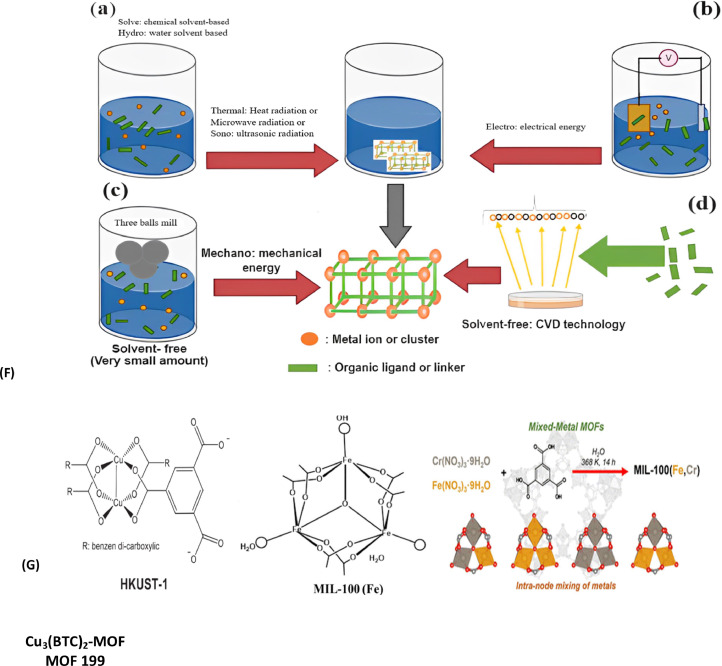
(A) shows the process of solvothermal synthesis for the structure of MOFs [10], (B) shows the microwave-assisted solvothermal synthesis process for the structure of MOFs (Modified from [10]), (C) shows Steps of MOF HKUST-1 synthesis by electrochemical method (a) SEM image of the product (b) (magnification, 20,000) Modified from [10], (D) shows Mechano chemical synthesis of MOFs [10], (E) shows Sonochemical synthesis of MOF structures [90], (F) Schematic representation of several MOF synthesis technologies: (a) hydrothermal, solvothermal, microwave-assisted, and sonochemical techniques; (b) electrochemical methods; (c) solvent-free mechanochemical method; (d) chemical vapor deposition, or CVD (solvent-free) modified from [91] and (G) The Cu_3_(BTC)_2_ metal-organic framework (MOF) (BTC = 1,3,5-benzene tricarboxylate), also known as HKUST-1 (or MOF199) [92].

Generally, several techniques are used for MOF synthesis. There are a few methods, which are commonly used for MOFs synthesis such as synthesis through room temperature, hydrothermal, solvothermal, microwave heating, mechanochemistry (MC), ultrasonic method (US), and electrochemistry (EC) as shown in Fig. 4 (A-E) [88]. Traditional synthesis of MOFs includes two methods, namely solvothermal and non-solvothermal methods. The solvothermal method is more common as compared to hydrothermal. Solvothermal uses any solvent whereas hydrothermal uses water only. Non-solvothermal synthesis follows the low boiling point of the solvent in an open flask at atmospheric pressure. On the other hand, solvothermal synthesis starts at the boiling point or above at a close flask and high pressure, which is formed by a pump or vapor produced by solvent. Commonly this method is used for the synthesis of MOFs [89] as shown in Fig. 4(A).

Synthesis through microwave is another technique used for MOF synthesis. According to a study presented by Hayes et al. ([93], microwaves produce electromagnetic radiation having a frequency from 300 MHz to 300 000 MHz. Among the two radiation components, magnetic and electrical, compounds can be synthesized by the former component only. Microwave radiation was used for MOF synthesis in 2005 for the 1st time [94]. The total synthesis time for MIL-100 (Materials of Institute Lavoisier) is reduced from 96 to 4 h with the help of this method. Hydrofluoric acid is an aqueous solution that is commonly used for the synthesis of MIL-100, trimesic acid (H_3_BTC), and chromium metal. All the reactants were mixed gently and kept in an autoclave, heated up to 20 °C by using microwave radiation [95]. According to [94], Cr-MIL-100 was the 1st reported MOF synthesized in this way exposed in Fig. 4(B).

The first report about the MOFs synthesized through the electrochemical method for HKUST-1 (MOF-199) was stated by Mueller et al. [96]. In electrochemical cells, copper plates are organized as anodes with the H_3_BTC liquefied in methanol as copper cathode and solvent. A precipitate of greenish-blue color was generated using a voltage of 12–19 V and 150 min (about 2 and half-hour). After the activation process, a powder with dark blue coloration powder obtained (crystals with an octahedral shape ranging from 0.5 µm to 5 µm in size) measuring 1820 m^2^/g of surface area as shown in Fig. 4(C).

Mechanochemical synthesis is another method for MOF synthesis. Mechanical energy is used in this reaction and at room temperature, the process of synthesis can be performed without the utilization of solvent [97]. According to [98], mechanochemical synthesis was first time used in 2006 for the synthesis of MOFs.

For a few minutes, they mixed (INA) isonicotinic acid and copper acetate in a ball mill. This method is directed to the production of fine-crystalized products along with the formula copper (II) isonicotinate dihydrate acetate (Cu (INA)_2_.xH_2_O.yAcOH). Acetic acid and water are both available in the pores of MOFs, and the results of the reaction lead to the formation of the products and can be eliminated by heat application Stock et al. [88] suggested that in a short reaction, the quantitative yield of MOFs can be obtained in 10–60 min Klimakow et al. [99] used a mechanochemical method in a standard ball mill with the assistance of liquid during the grinding process, Powder of (H_3_BTC) 1,3,5 benzene tricarboxylic acid and copper acetate monohydrate as well as (H_3_BTB) 4,4′,4′’- benzene tri-benzoic acid were used separately. Furthermore, 3:2 was kept as the molar ratio and the ball mill process lasted for 25 min. Synthesis through the mechanochemical process is only limited to a particular type of MOFs and obtaining a huge amount of product is very challenging through this method. HKUST-1 (MOF-199) was produced successfully without the use of solvent by the mechanochemical synthesis method, as shown in Fig. 4(D) [100].

The sonochemical method is also included in the MOF synthesis method. The effect of ultrasound on both the colloid system and liquid is mostly produced by cavitation. This is all about air release and vapor formation, which is led by a pressure decrease in liquid as a great-strength acoustic wave, spreads through it Qiu et al. [101] reported that the sonochemical method was used for the 1st time for MOFs (Zn_3_(BTC)_2_) synthesis in 2008. Materials like zinc acetate and (H_3_BTC) 1,3,5-benzene tricarboxylic acid are mixed with 20 % ethanol and made ready for the process of sonication for a few minutes about 90 min (about 1 and a half hours). But after 5 min of the sonication process, a high amount of product was achieved (75.3 %). Son et al. [102] used the sonication method to decrease the time of MOF synthesis from 24 h (conventional heating) to 75 min. Synthesis was made like terephthalic acid with 1-methyl-2-pyrrolidone and a solution of zinc nitrate was mixed well in a nitrogen atmosphere. Subsequently mixture was added to the sonication process for 10–75 min after transferring into a reactor. MOFs-5 precipitation begins after 8 min of the sonication process. Furthermore, this method can be used for Cu_3_(BTC)_2_ (HKUST-1) synthesis by using H_3_BTC solution in a Dimethyl formamide (DMF) mixture with ethanol and copper acetate solution [103] as presented in Fig. 4(E).

The procedure of crystallization also involves MOF synthesis where crystal growth and nucleation occur. Self-assembly is included in between the metal oxygen clusters and organic linkers for the MOF crystal growth and nucleation process. Crystal morphology and MOF sizes will be carefully controlled when the influence factors of the MOF growth and nucleation are fully understood. Besides all this, it is commonly known that a very important character is played in the size and morphology of MOFs by many factors such as synthesis conditions of MOFs such as solvent type, time, concentration of reactant, and temperature [104]. According to Desiraju in 2007, crystallization can be considered as supramolecular reaction. Erdemir et al. [105] and Schuth et al. [106] suggested nucleation and growth as a two-step process for the crystallization of MOFs.

Davey et al. [107] defined nucleation, as when dispersed nuclei form from a homogenous solution under supersaturating is known as nucleation. The solid phase is separated from a solution when a critical nucleus is formed. Liquid-like clusters, aggregate (supramolecular transition state), and high energy transitional are represented by critical nuclei to act as a link between the solid and solution. According to Saha et al. [108], the crystallization of MOFs and the states preceding crystallization impact the nucleation rates. Information about the crystal's size is hard to obtain at the early stages of crystallization. For the time-resolved and characterization study of solid-state structure, Perrin et al. [109] proposed analytical probes, such as Fourier transform infrared spectroscopy (FT-IR), mass spectrometry, and nuclear magnetic resonance (NMR). However, it was always discovered to be deficient in critical nuclei or intermediate characterizing.

## 3 Characterization of MOFs

Various physiochemical methods are used to study the properties of different MOFs synthesized through different methods. It is essential to know about the textural and structural MOF properties and study their stability and homogeneity. Several changed procedures were used to characterize the MOFs such as Transmission Electron Microscopy (TEM) and Scanning Electron Microscopy (SEM).

**Fig. 5:**
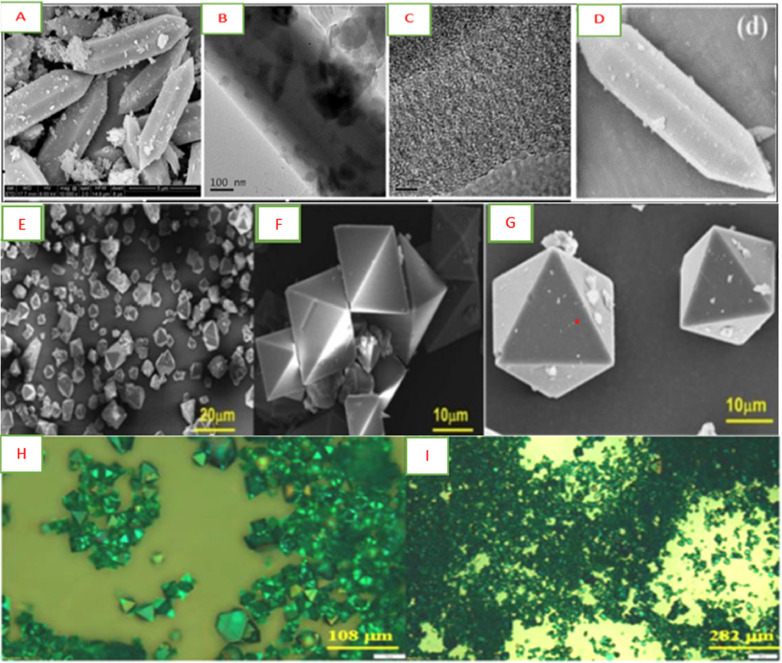
(A) Image of an iron-based metal-organic framework (Fe-MOFs) obtained through (FE-SEM) Field Emission Scanning Electron Microscopy (B) Fe-MOFs images obtained through (TEM) Transmission Electron Microscopy (C) Fe-MOFs images obtained through (HRTEM) High- Resolution ТЕМ, and, (e-g) (EDX) Energy Dispersive X-ray Analysis element mapping of O, Fe, and C found from (D) [110], (E, F, and G) At different magnification images of (Cu-BTC MOFs) Copper (II) Benzene-1,3,5-tricarboxylate MOFs obtained through FE-SEM (H and I) Cu-BTC MOF's optical microscopic images at various amplifications [112].

The SEM techniques are widely used for the characterization purposes of MOFs. According to Gao et al. [110]; and [111], two-dimensional images with high resolution are produced by scanning electron microscope techniques, which are used for MOF characterization. The shape of the materials, revealing information about external morphology, their spatial variations, mixing, and dispersion of phases are displayed by it. The structure is shown by MOFs. Particles of different shapes created by MOFs show different shapes such as bars, rhombohedral, and cubes, and produce a different morphology as mentioned in Fig. 5 (A-D).

According to the authors, the characterization of SEM mostly required platinum and gold for the surface coating having a conductive material, which is based on the insulating nature of MOFs. To avoid this problem, the researchers got access to (FESEM) Field Emission Scanning Electron Microscope. The field emission gun is used in this method with which the equipment works that delivers a great focused electron beam. The spatial resolution work is enhanced by this method or technique at a very low potential. The charging effect is also reduced by these characteristics on the insulating materials and damage is avoided even though a beam of an electron can be prompted in about different sensitive types of MOFs.

These beneficial techniques can be related to energy dispersive X-ray analysis (EDAX or EDX) or energy dispersive spectroscopy (EDS), which permits for defining of the MOF's qualitative compositions and elemental analysis. Fe-MOFs were synthesized by [110], where its FE-SEM images are shown in Fig. 5(A), and its spindle-like uniform shape is expressed. This observation also represents the successful linking and reaction of all the reactants, resulting in the development of iron-based MOFs (Fe-MOFS) within the specific system of reaction. Furthermore, (FE-SEM) field emission scanning electron microscope, as well as (HR-TEM) high-resolution transmission electron microscopy, characterizes a constant axis-like shape. Similar lattice structures and microstructure are shown in Fig. 5 (B and C). EDX-yielding mapping results were used to study the chemical compositions of each element. This allowed us to know the similarities of the MOFs synthesized already where the constant existing elements of O, Fe, and C in the structure of iron-based metal-organic framework (Fe-MOFs) are confirmed, suggesting its possibility to be used as a template for the production of adsorbent based on iron (Fe). EDX mapping is shown in Fig. 3(D).

SEM is a useful tool for learning about a variety of MOF attributes, including crystal size, morphology, and elemental composition, as we have previously covered. Depending on the size of the MOF crystals, optical microscopy may also be used to get basic information on the morphology and crystal size. Another example given by Kim et al. [112], presented the textural structure and morphology of the Cu-BTC MoFs. (FE-SEM) Field Emission Scanning Electron Microscopy further explored this. Their optical analysis is shown in Fig. 5 (E-I). The images show that the prepared MOFs hold a cubic crystal structure characterized by octahedral geometry where the length for all the edges separately of octahedral is recorded as approximately 10–20 μm.

Transmission Electron Microscopy is a beneficial procedure. It has been used commonly to define the size of particles and grains and crystallographic data including indices and plane dislocation. The microscopic images can be analyzed by using different software programs such as (imageJ and Cell Profiler). Generally, different particle sizes have been determined by this method beside all this crystallographic data has been created with histogram. This is an advantageous technique for MOF characterization changed by the integration of nanoparticles. Data about the dispersion and size of those nanoparticles are presented by the obtained images [113].

**Fig. 6:**
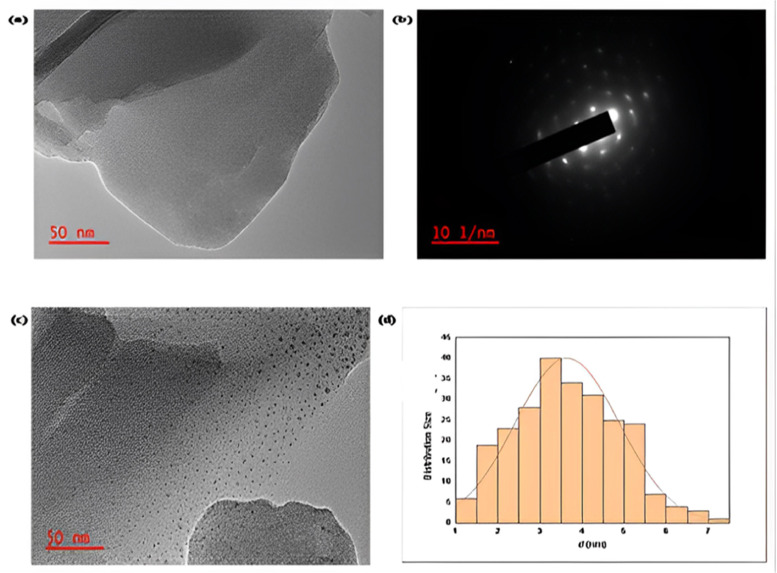
(a) Image of Cu-MOFs obtained before catalysis through (HR-TEM) High-Resolution Transmission Electron Microscopy at 50 nm scale, (b) Insert presenting (SAED) Selected area (electron) diffraction pattern for Cu-MOF, ( c) Cu-MOFs images obtained after catalysis through HR-TEM (4th cycle) at 50 nm scale and (d) Histogram of the distribution of the particle size for Cu-MOFs after analysis (4th cycle) [114].

Patil et al. [114] presented the images of copper metal-organic frameworks (Cu-MOFs) obtained through (HRTEM) High-Resolution Transmission Electron Microscopy presented in Fig. 6(a). The crystalline nature of Cu-MOFs is confirmed by the (SAED) Selected area electron diffraction extra shown in Fig. 6(b). HR-TEM images were collected mostly after catalysis to check the Cu-MOF development as mentioned in Fig. 6(c). The spread copper (Cu) was noted on the spent Cu-MOF consistently with a standard deviation of ± 1.3 nm. The particles with unchanging average size each having a 3.5 nm diameter are given in Fig. 6(d).

The above discussion was about the different characterizations of MOFs which are discussed with examples with many methods such as TEM and SEM but depending on the MOFs based materials and for the purpose the required MOFs are used should be done with different methods. The MOFs prepared with different methods need to be characterized with many physiochemical methods for defining their properties related to each one. From the perspective of water treatment point through photocatalysis, to knowing much more about water stability, their textural and structural properties are very important. The most useful techniques for characterization are given below.

The wide use of (PXRD) powder X-ray diffraction can determine the degree of structural and crystallinity parameters of MOFs. Qin et al. [115] stated that the identification of structure can be completed when a MOF is created and their diffractogram is related to the previous one which is documented in the literature or explained through a replicated pattern obtained through only crystal X-ray archived through computational modeling or in a database. These techniques help identify the crystalline structure, change the polymorphic form, and differentiate between crystalline and amorphous materials to find the estimated percentage of crystallinity. When the MOF structure is confirmed and recognized as crystalline, then the determination of crystallographic parameters is possible, for example, the unit cell size, size of crystallite, and lattice parameters. The formers and diffraction data can be distinguished by applying various methods where a mathematical alteration is accepted, for example, the method of non-linear least squares [116]. Once the peaks of diffraction are identified, Scherrer's equation is used to calculate the crystallite size commonly where no overlapped peak with the most intense [117].

$$D=\frac{K.\lambda }{\beta cos\theta }$$

The size of the crystallite is represented by *D*, and the factor size is represented by *K*, which can vary depending on the equipment's characteristics. The full width at half-maximum height (FWHM) of the peak is indicated by *β*, and the Bragg angle matching to the peak found in the diffraction pattern is indicated by θ.

**Fig. 7:**
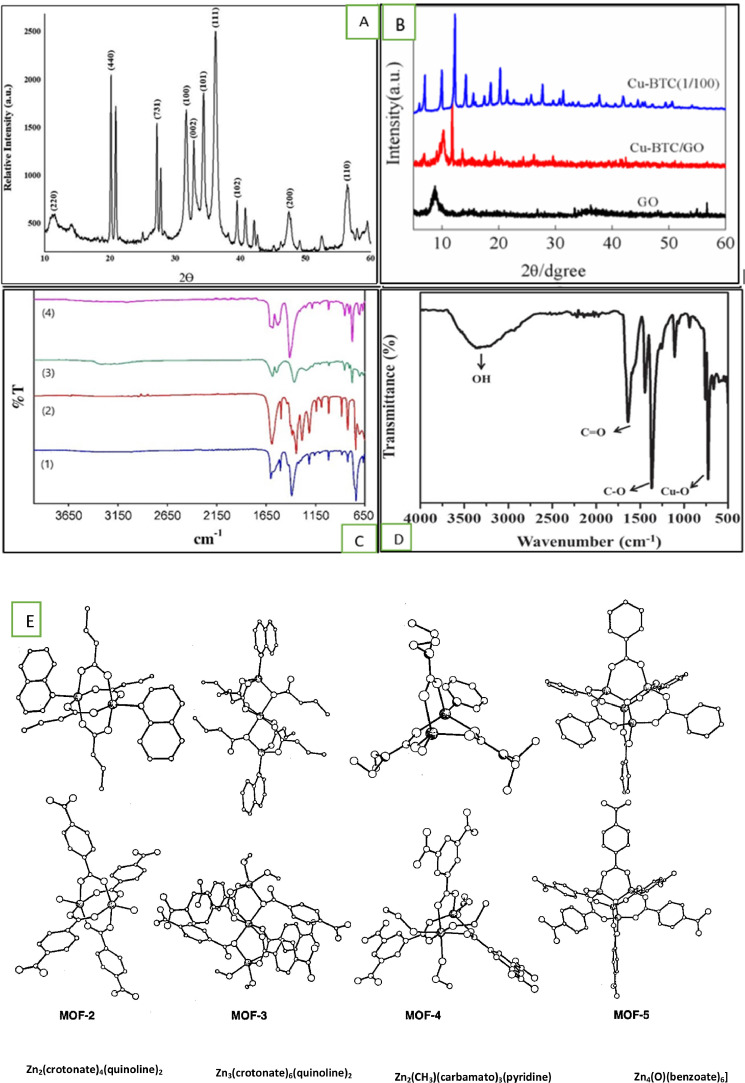
(A). Powder XRD diffraction illustrating the synthesized zinc ion Benzene-1, 3, 5-tricarboxylate MOFs (Zn-BT) ([120,121]; and [122]). (B) (XRD) X-ray diffraction analysis for (Cu-BTC) copper 1,3,5-benzene tricarboxylic acid, GO/ Cu-BTC, and Graphene oxide (GO) [123]. (C) FT-IR spectra of pristine MOF-5 (1); pristine MOF-177 (2); NH_3_ showing MOF-5 (3); NH_3_ showing MOF-177 (4) [124], (D) FTIR spectrum of Cu-BTC MOF [125], and (E) An overview of the Zn-O-C cluster SBUs' structural relationships with discrete metal carboxylate clusters [39]. *Atoms are shown as spheres with Zn, shaded; O, large open; N, partially shaded; C, small open. Hydrogen atoms have been omitted for clarity.

This procedure allows us to understand the full characterization of MOFs, which is why fully used by the research working on MOFs as well as the creation of new MOFs, composite or heterojunctions are also served by it. In applications of photocatalytic, the XRD techniques also control the constancy of the MOF. After the reaction, the used MOFs are recovered, subjected to the washing procedure, and dried then, after processing the diffraction pattern is documented. To check the possibility of differences and determine whether the MOF's structure is constant or not, the processed MOF is compared with the pristine MOF [118]. One most common methods for the preparation sample of a (PXRD) powder X-ray diffraction is to load the sample into powdered form in a flat plate holder sample that is usually made up of glass, aluminum, or plastic. Samples should be dry or affixed using oil or volatile solvents loaded onto the holder. This method is suitable for most of the samples as shown in Fig. 7 (A-B). The MOFs should be rotated continuously during the collection of data (in a capillary tube or sample holder) to avoid issues regarding preferred orientation [119].

The PXRD method is used to regulate the main crystallinity of MOF samples. An example is given by Ayub et al. [120,121]; and [122] where they presented the recorded PXRD diffractogram for the Zn-BTC as shown in Fig. 7(A). The recorded diffraction peaks on 2ϴ scale are 56.59°, 47.56°, 40.81°, 39.42°, 36.28°, 32.90°, 31.80°, 27.22°, 20.15°, and 11.36° resembles indices of miller (110), (200), (102), (111), (101), (002), (100), (731), (440), and (220). Each of these peaks aligns with the pattern of standard diffusion pattern where face-centered cubic (fcc) crystals for Zn-BTC were observed with the minor shifting of peaks because of matching guest molecules.

Sajjadifar et al. [126] reported about the Zn-BTC and calculated its average crystallite size by recording the X-ray diffractogram using a formula of Scherrer given below.

D=0.89λ/βcosΘ

The X-ray wavelength is represented by λ, the Bragg diffraction angle by 2ϴ, and the full width at half maximum (FWHM) of the high diffraction peak is represented by β. The average crystallite size of Zn-BTC is estimated to be 5.96 nm, although the degree of crystallinity was found to be 79.03 %. [[50], [127]] provided another illustration of the PXDR of Cu-MOFs. While the two other samples were created by depositing the distributed resources onto the substrates, the Cu-BTC sample is a powder. Cu-BTC exhibits a significantly greater XRD intensity in comparison to the other two samples consequently. Cu-BTC's X-ray diffraction (XRD) intensity has been set to 1/100 for comparison's sake. Observationally, the powdered samples accurately depict the unique properties of Cu-BTC.

There was a good level of crystallinity in the synthesized product when strong peaks showed up in the XRD pattern. Fig. 7(B) illustrates the normal range of 8–11° for the peak of GO [123]. Bragg's law indicates that the primary peak of GO, which was measured in our experiment at 2θ = 8.59°, suggests a 10.3 Å gap between the carbon layers. The deposition of Cu-BTC on GO causes GO to become the 2θ = 10.17° (8.8 Å) indicated by the peak. Graphene oxide (GO) and Cu-BTC are found to have peak features in the extra Cu BTC/GO (XRD) X-ray diffraction pattern.

As compared to previous commonly used techniques, one main development of FT-IR is multiplex advantage where the analysis of all the present wavelengths is allowed at the same time instead of consuming the monochromatic radiation [128]. An example is given by Petit et al. [124] where FT-IR spectra of MOFs-177 as well as MOFs-5 are shown in Fig. 7C (1–4). In MOFs-5, the unequal widening of the carboxylate group of BDC is shown in Fig. 7C (1) in around 1501 cm^−1^ and 1588 cm^−1^, and equal widening seems to be about 1388 cm^−1^ of the identical material. Some bands within the range of 1284 cm^−1^ - 730 cm^−1^ are detected and these can be recognized as the out-of-plane benzene dicarboxylate (BDC) vibration. In FT-IR of NH_3_ showing MOF-5 in Fig. 7C (2), the highest positions continued nearly matching with changes no more than ±5 cm^−1^ for limited peaks. The major alteration found among the top of NH exposed and pure MOF-5 is that around is a high range rise in top intensities of all the top peaks in the spectrum of NH_3_, which are exposed to MOFs-5. Fig. 7E presents the Zn-O-C cluster SBUs' structural relationships with discrete metal carboxylate clusters [39].

For MOF-177 shown in Fig. 7c (3), shows that the peaks for MOFs-177 are considered to be similar due to the availability of similar types of organic acid (BTB). The irregular widening, regular widening, and out-of-plane vibrations seemed at approximately 1536 cm^−1^–1581 cm^−1^, 1360 cm^−1^, and 1231 cm^−1^–700 cm^−1^. The FT-IR spectrum of MOF-177 where the alteration made visible to NH_3_ is shown in Fig. 7C (4) seems to be rather parallel to that of MOF-5 exposed to NH_3_, the top positions are mostly not changed with a less increase in high strength.

The Meso-Cu-BTC Metal-organic framework's molecular configuration may be examined using FTIR, as demonstrated by the following example [129]. According to Ganesan and Lee [125], Fig. 7(D) displays the bands at 720 cm^−1^ and 480 cm^−1^ that correspond to the vibrational and stretching modes of Cu-MOFs. Vibration bending of the aromatic ring's in-plane and out-of-plane movement is responsible for the tiny peaks located between 660 cm^−1^ and 760 cm^−1^. The regularly occurring and asymmetrically occurring O—C=O and C—O extending vibrations of molecular moieties are responsible for the band seen between 800 cm^−1^ and 1150 cm^−1^. Additionally, prominent peaks of absorptions are known on 1375 cm^−1^ along with 1432 cm^−1^ as well as 1625 cm^−1^.

The vibrational modes of the (COOH) carboxylate group in Meso copper 1,3,5-benzene tricarboxylic acid (Meso-Cu-BTC) might be the source of these signals. They show similarities to the individual C=O and C—O symmetric and asymmetric stretching modes. The carboxylate group's association with copper ions (Cu ions) is what is thought to be responsible for this resemblance [130,131]. The water coordination in the Cu-MOFs is responsible for the band of absorption at 1542 cm^−1^. Meanwhile, the water-adsorbed surface and hydroxyl group (OH group) in the Meso-Cu BTC metal-organic frameworks are linked to the peaks seen at 3390 cm^−1^. Additionally, Meso-Cu-BTC MOF formation is supported by the catalyst's peak occurrence at 1719 cm^−1^, which reveals the presence of benzene tricarboxylic acid.

The robust framework is prepared to use carboxylate linkers such as 1,4-benzene dicarboxylate (BDC) and 1,3,5-benzene tricarboxylate (BTC), by giving an advantage because of their rigidity and tendency by forming a metal carboxylate clusters that eventually perform as secondary building units (SBUs). The zinc (II) carboxylate clusters have been prepared by using BDC and BTC as linkers to give Zn (BDC)‚(DMF) (H_2_O) (DMF) N, N′-dimethylformamide) (MOF-2), Zn_3_(BDC)_3_.6CH_3_OH (MOF-3), Zn_2_- (BTC)(NO_3_). (C_2_H_5_OH)_5_(H_2_O) (MOF-4), and Zn_4_O(BDC)_3_. (DMF)_8_(C_6_H_5_Cl) (MOF-5). The structures are presented in Fig. 7E (Eddaoudi et al., 200).

## 4 Antibacterial activities and drug delivery of MOFs

The following factors are noted to be responsible for the MOFs' antibacterial activities, according to Livesey et al. [132] who examined the various pathways based on their functionalities. MOFs slowly release antimicrobial agents present inside them, acting as storage, and many loaded antimicrobial molecules are released which are frequently held inside the MOFs through supermolecule forces. Bioactive MOFs are depredated as well, and linkers/metal ions are released. The availability of photosensitizer molecules shows photo activity is responsible for acting as a chelating agent and physical disinfection.

Generally, drugs are loaded in MOFs in a post-synthetic way. In a pre-synthetic method, MOFs are filtered out after stirring in drug solutions. The major benefit of this procedure is that by changing the time of stirring and concentration, the drug amount can be controlled. However, some MOFs can be disintegrated during the post-synthetic method Velásquez-Hernández et al. [133]. Three cargo loading strategies—encapsulation, direct assembly, and post-synthesis strategy—were reported by Wang et al. [134] for MOF loading with substantial drug concentrations. The drug's position was taken into consideration when determining the strategic categorization.

**Fig. 8:**
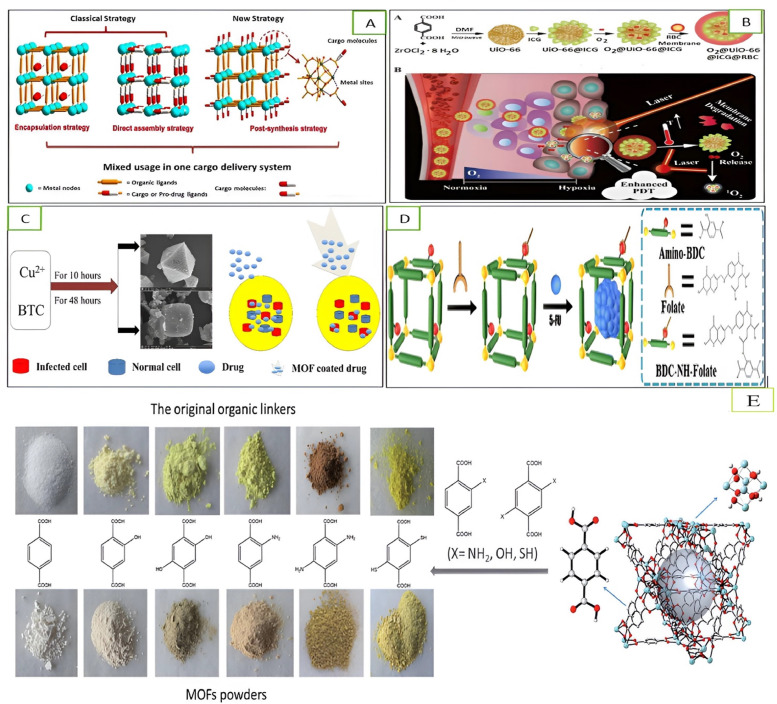
(A) MOF's loading strategies of cargo such as post-synthesis, encapsulation, and direct assembly [134]. (B) (a) O_2_@UiO-66@ICG@RBC synthesis (B) Enhancement of NIR-triggered O_2_ and PDT discharging by schematic mechanism [110]. (C) Synthesized HKUST-1 with cubic and octahedral morphology at different intervals (10 hrs and 48 hrs) and drugs are delivered by drugs-coated MOF nanoparticles to the targeted tissues without disturbing other cells [138], (D) Schematic representation of the encapsulating 5-fluorouracil 5(FU) in FA-IRMOF-3, and (E) Structure of UiO-66 (color/atom: gray/C; red/O; white/H; light blue/Zr); left: the photographs of UiO-66 with various organic ligands and its original precursor linkers.

In an encapsulation strategy, the cargo resides inside the pores or channels of MOFs through non-covalent bonding interaction. The MOF's structure is not changed by this type of strategy. A wide series of hydrophobic, amphiphilic, and hydrophilic molecules of drugs were enclosed in the MOFs. The exploration of MOF functionalization reveals several study fields concerning pharmaceutical and biological applications, except the attachment of therapeutic molecules on MOF surfaces. To successfully deliver drugs to the target, the necessary modification must, however, not only increase MOF stability and reduce interaction with the biological medium but also make it easier for drugs to pass through physiological barriers ([135,136] and [137]). A nanoplateform was constructed by Gao et al. [110] with prolonged circulating properties. MOF-based zirconium (UiO-66) was used as a carrier for O_2_ storage. In the first step, UiO-66 is linked with (ICG) indocyanine green by a coordination reaction, which is followed by surface through encapsulation with (RBCs) red blood cell membranes as shown in Fig. 8(b).

Direct assembly strategy involves the coordination bond formed between the cargo and MOFs. The cargo contributes as a ligand in synthetic reactions to partially contribute to the MOF's structure. Magnetic nanoparticles of MOFs perform actions of drug delivery systems directing to the current location of treatment. In this context, [138] produced flexible and porous copper-based MOFs (Cu-MOFs). The compound Cu-benzene tricarboxylic acid was designated as HKUST-1. Cu-BTC's crystalline development has been optimized by the application of hydrothermal methods. The synthesis procedure involved the use of nontoxic solvents. As shown in Fig. 8(c), the absorption of the medicine paracetamol was measured using HKUST-1 at 10 and 48 h (about 2 days). The cargo molecules are present on MOF surfaces in the post-synthetic method. These molecules work as a linker to help pre-synthesized MOFs come together. The development of covalent connections and coordination bonds between the metal nodes and organic linkers, as well as the used cargo, are examples of the chemical interactions involved in this technique. The structure of MOFs is not changed by this type of strategy. Adsorption is the second possibility of this strategy on the MOF surface Yang et al. [139] synthesized (FA-IRMOF-3) folate-targeted zinc-based nanoMOFs by using a post-synthetic strategy to check out the FA-IRMOF-3 performance as a tumor cell-targeted drug carrier. FA-IRMOF-3 is prepared through the conjugation of folate with zinc, which is based on nanoMOFs shown in Fig. 8(D). Many malignant tumors express high levels of folate receptors on their surface. Therefore, folate was chosen as the targeting ligand for nanoMOFs designed to deliver the anti-cancer drug 5-fluorouracil (5-FU) to tumor cells. The effectiveness of this approach was tested in three different cancer cell lines: those with high folate receptor expression, those with low expression (Hela cells), and those without expression (A549 cells). Additionally, it was compared the cytotoxicity of 5-FU-loaded nanoMOFs to direct exposure of 5-FU in these cancer cells.

MOFs can be loaded in concerned drugs in a controlled environment for the better result. Antimicrobials can be used as MOF components like an ion or metal. For example, in the year 2019, nalidixic acid, which is 1st generation quinolone and a broad-spectrum antibiotic used as a linker in manganese (Mn) and magnesium (Mg) base MOFs [140]. Another mechanism of antimicrobial action is physical disinfection. Many physical disinfection methods have been used to provide a neat clean environment from microorganisms and microbial growth can be stopped by preventing their attachment to the surface.

Furthermore, metal oxides including Cu_2_O, ZnO, CoO, NiO, and CuO are considered to be very active antimicrobial agents, especially in the nanoform. They are semiconductors and their activities against bacteria have been recognized for (ROS) reactive oxygen species formation [7,8]. The study concludes that, as the tables below illustrate, various MOFs and their derivatives are the best options to employ as antimicrobial agents in various contexts.

Two strategies are used generally for MOFs to absorb light accounting for more than 42 % of solar light radiation. Different ligands were used such as H_2_BDC, H_2_BDC—OH, H_2_BDC-2,5OH, H_2_BDC—NH_2_, H_2_BDC-2,5NH_2_, and H_2_BDC-2, 5SH, individually. These MOFs had strong light absorbent capacity and exhibited different colors as shown in Fig. 8E.

Bacterial viability kits were used to stain all cells, dead cells of bacteria can be identified by cell membranes that become damaged and were labeled properly with propidium iodine, and red nucleic acid whereas all cells of bacteria were labeled by using SYTO 9 (Green). Red and green regions individually represent the live and dead cells with scale bars set at 100 µm [141].

**Fig. 9:**
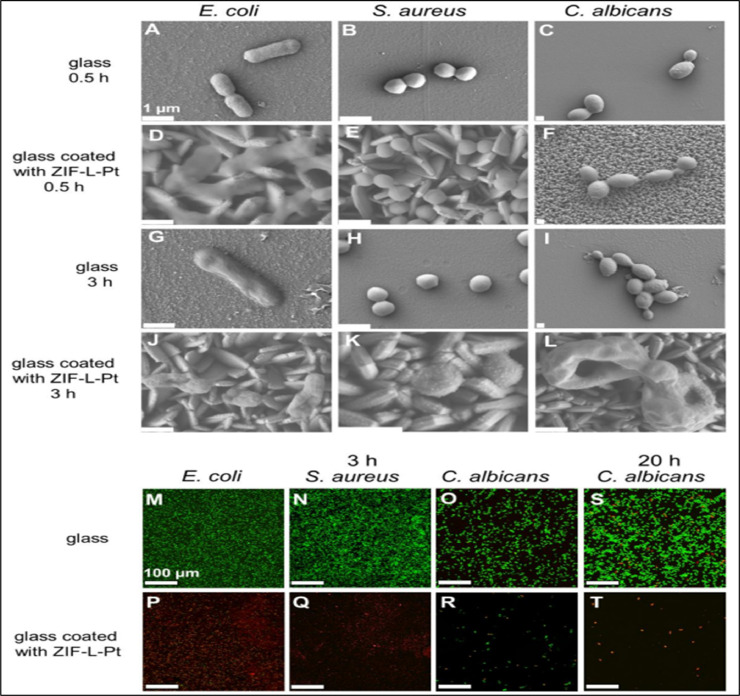
Cell capability and morphology were examined in both coated and uncoated ZIF-L glass surfaces. SEM images (A-L) depict *S.aureus, C.albicans,* and *E. coli* cells on (A-C, G-I) uncoated besides ZIF-L-PT (D-F, J-L) coated glass were observed after 0.5hours and 3 h of incubation with scale bar set at 1 µm. Images of a confocal laser scanning microscope (M-T) showed *E. coli and Staph. Aureus* and *Candida albicans* cells on (M-O, S) uncoated and ZIF-L- Pt- (P-R, T) coated glass can be observed after an incubation period of 3 h and 20 h.

According to [141], leaf-shaped crystals of zeolitic imidazolate frameworks (ZIF) were created by substituting a ratio of (Zn^2+^) metal for the ligand (2-methylimidazole or 2-melm). ZIF-coated surfaces are germ-free and exhibit antimicrobial activity against a variety of pathogens, including gram-positive and gram-negative bacteria including *Candida albicans, E. coli*, and *Staph. Aureus*. Additional studies were conducted to determine the additional antibacterial mechanisms—chemical interactions and Zn^2+^ leaching—that contribute to this result. Using a scanning electron microscope (SEM), microbes exhibiting morphological alterations were detected when they came into touch with surfaces coated in nano-daggers. The results demonstrated that all bacteria were deformed in three hours and that all cells were killed in twenty hours. This kind of communication is only physical. As seen in Fig. 9, the negatively charged bacterial cells electrostatically interact with the nano-dagger-shaped ZIF, resulting in structural deformation. Fig. 9 (A-L) illustrates the adherence of three microbes to ZIF-L uncoated and coated glass at contact timings of 3 h and 0.5 h. After an incubation period of 0.5 h on ZIF L- PT, *E. coli* was found compressed but morphology was detected normal, due to the thick and rigid cell wall of *S. aureus* their morphology was unable to be observed at 0.5 h. All three cells started deformation after 3 h. Cells of *E. coli* and *C. albicans* were found ruptured. After an incubation period of 4 h, nano daggers penetrated the cell wall of *C. albicans* can be observed. The *S. aureus* and *E. coli* can be seen stained red mostly in Fig. 9 (P and Q). This is after the rupturing of the targeted bacterial cells with ZIF-L-Pt coating after 3 h of incubation. Some *C. albicans* cells were ruptured after three hours of incubation period with ZIF L-coating whereas some cells were still alive, as can be noticed in Fig. 9(R). However, no live cell can be seen after 20 h of incubation period as shown in Fig. 9(T).

Table 3 shows different MOFs and MOFs derived materials with activity against different bacteria with different mechanisms of action. It shows the different MOFs-based materials showing their best antibacterial response; mostly the targeted organisms are *P. aeruginosa, MDR-P. Aeruginosa, B. substillis, E. coli,* MRSA and *Staph. aureus* with different mechanisms of action and MOFs-based materials and zones of inhibition.

**Table 3:** Different MOFs and MOFs derived materials with activity against different bacteria with different mechanisms of action.

MOFs and MOFs based materials	Antibacterial Agent	Mechanism of Action	Targetted bacteria	Antimicrobial Efficacy	References
Silver-MOFs (Ag-MOF)	Organic radical anions Ag+	MOFs breakdown photochromism	*Multidrug Resistant S. Aureus* *Pseudomonas aeruginosa* *MDR-P. aeruginosaE* *. coli* *Bacillus. subtilis* *Staph. aureus*	*In vitro*: inhibits drug-resistant microorganisms over 98.47 % of vivo: Healthier healing of Multi drugs resistance *P. aeruginosa* infected wounds in mice injury	[71]
Copper-MOFs (Cu-MOF)	Reactive oxygen species (ROS)	Photodynamic therapy uses light and Photosensitizing agents to kill m/o and abnormal cells	*E. coli* *and* *Staph. Aureus*	*In vitro*: As lowest concentration (Minimum Inhibitory concentration) MIC: 300 µg/mL MIC: 350 µg/mL	[142]
Cu-MOFs	Reactive oxygen species (ROS)	Using Chelation effect	*E. coli* *Staph. aureus* *Klebsell. pneumonia* *MRSA* *Pseudomonas aeruginosa*	*In vitro* Minimum bacterial concentration 20 µg/mL	[143]
Zeolitic imidazolate frameworks (ZIF-8)	(Chloramphenicol) Specially used in eye conjunctivitis caused by bacteria	Release of drug	*S. aureus* *E. coli*	Reduce more than 99.9 % bacterial growth in 24 h.	[144]
Zeolitic imidazolate framework 8 (ZIF-8)	Ceftazidime	Drugs releasing	*E. coli*	Experienced with less bacterial growth after being exposed to MOFs	[19]
ZIF-8	Gentamicin	Releasing for drugs	*S. aureus* *E. coli*	Zone of inhibition recorded <14 mm 12mm	[145]
ZIF-8	ciprofloxacin	Releasing for drugs	*S. aureus* *E. coli*	Zone of inhibition recorded 46 mm (about 1.81 in) and 49 mm (about 1.93 in)	[146]
Cu-MOFs nanoparticles	Fe3+ Ferric ion	Morphology of Chelation Particle	*Staph. aureus* *Klebsiella spp.* *Candida spp.* *E. coli* *Pseudomonas spp.*	Zone of inhibition recorded (100 µg/ml concentration) 42, 46, 45, 49 and 35 mm (about 1.38 in)	[147]
zinc (II) benzene dicarboxylate Zn-BTC	Kanamycin and Ampicillin	Release of Drugs	*Staph. aureus* *E. coli* *Staphylococcus lentus* *Listeria monocytogenes*	Improved antimicrobial results against gram-positive and gram-negative bacteria	[148]
(Co-SIM-1) Cobalt imidazolate-MOFs	Silver Tetrazolate Aggregation Zone (AgTAZ)	Antibacterial activity and shows durability with materials	*P. putida* *E. coli* *S. cerevisiae*	Average zone of inhibition recorded 15 mm	[44]

## 5 Antifungal activities of MOFs

MOFs were recognized for their diverse antimicrobial properties, extending beyond antibacterial effects to encompass antifungal and antiviral activity. Researchers have extensively investigated the potential of silver-based MOFs to combat fungal infections. Studies by Alisir et al. [149] identified [Ag_4_(μ-pydc)_2_(μ-pm)_2_]_n_, a polymeric silver complex, as effective against Candida albicans (C. albicans). Similarly, Jaros et al. [150] demonstrated the strong antifungal activity of Ag(I) MOF23 against C. albicans. Furthermore, Sheta et al. [147] explored the antifungal properties of copper glycinate (Cu(II) MOF-29), highlighting the diverse possibilities within MOFs for tackling fungal infections. Cu-MOF 29, which was based on 2, 3-diamino-5-bromopyridine, and 5-amino isophthalic acid, was found to have increased antifungal efficacy against *Candida albican*. The best options for antibacterial agents against the same species of Candida albicans are MOFs-36 and 60, which are based on 4,40-bipyridyl and 3-phenyl-1H-pyrazole-4-carboxylate linkers, Co (II) and Ni (II).

Zn (II)-MOFs 42, which is based on 3′-(1H-tetrazol-5-yl)- [1,10 -biphenyl]−4-carboxylic acid, shown strong antifungal action [151]. In addition to Zn (II), Ni (II), and Cu (II)-MOFs, which are only reported to be effective against *Candida albicans*. Cyclodextrins MOFs (Cd (II) MOF 50) have been shown by Miodragović et al. [152] to be effective not only against the fungus *C. albicans* but also in preventing the growth of *Aspergillus niger* (*A. niger*).

According to a study presented by Karadağ et al. in 2015, heterometal dicyanidoaurate (I) MOFs 67–71 were found to be effective against a variety of fungi, including *Alternaria solani* (*A. solani*), *Rhizoctonia solani* (*R. solani*), and *Fusarium oxysporum f.sp. lycopersici* (*F. oxysporum*), which is typically thought to be a plant pathogenic fungus. According to a study conducted in 2019 by Kim et al., cells of *Candida albicans* were deactivated more effectively than those of *A. niger* by Co(II) MOFs containing linkers like bipyridyl and glutarate [Co_2_(Glu)_2_ (bpe)}(H_2_O)0.5]_n_ (137), [Co_4_(Glu)_4_(bpp)_2_]_n_ (144), and [{Co_2_(Glu)_2_(bpa)}(H_2_O)_4_]_n_ (143).

Chiericatti et al. [153] investigated Cu (II) MOFs (HKUST) as an antibacterial agent used against fungi. It was noticed that the growth rate of *Geotrichum candidum (G. candidum)* was reduced strongly and the growth rate of *Saccharomyces cerevisiae (S. cerevisiae)* was completely inhibited. This antifungal activity is linked with the release of Cu^2+^ ions. These ions bind to the fungus cell wall and disturb nutrient transportation and intracellular enzymes are inhibited.

**Fig. 10:**
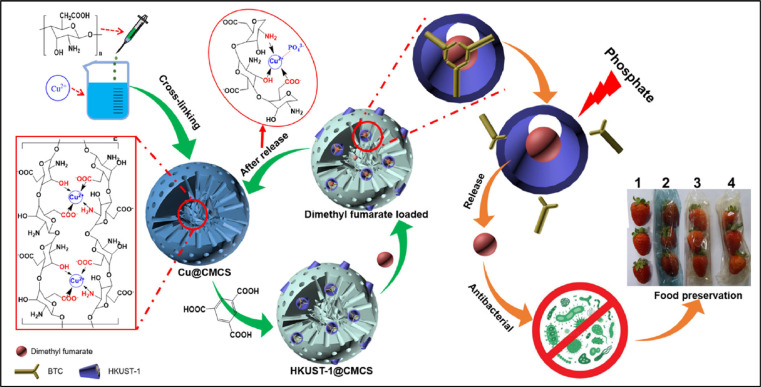
HKUST-1@CMCS production, regeneration, phosphate-stimulated drug release without Cu (II) ion residues, antibacterial activity, and food preservation of dimethyl fumarate-loaded HKUST-1@CMCS film.

To create an environmentally friendly, recyclable, long-acting intelligent antibacterial vector, HKUST-1@CMCS, a novel method of mutual encoding of carboxyl chitosan (CMCS) and HKUST-1 was presented in Fig. 10. The thorough characterization demonstrated that the structure of HKUST-1@CMCS was gradually disrupted at varying intensities by phosphate stimulation, resulting in the intelligent release of antibacterial medications.

Different bioactive molecules are linkers with different antifungal MOFs [140] concluded that Mn (II) along with Mg (II) MOF (MOFs 74 and 75) based on nalidixic acid found useful against different yeast strains for example *S. cerevisiae* and *C. albicans.* Another study about the antifungal MOFs and their linkers was presented by Hamamci Alisir et al. [154]. Antifungal activity against *C. albicans* is exhibited by Ag (1) MOFs 87, which contains 2,5- dimethylpyrazine and diclofenac Abdelhameed et al. [155] showed that Zn (II) based MOFs 40, 93 with 94 were found beneficial against different fungi including *Cerevisiae, A. niger, C. albicans, P. verrucosum, F. proliferatum A. parasiticus* and *A. flavus.* Table 4 summarizes the various types of MOFs with their linkers, compositions, fungus tested, and the results. Many MOFs showed excellent results inhibiting or reducing the growth rate by about 99.99 %.

**Table 4:** Antifungal activity of MOF composition and linkers, MOFs, or composites were tested and tested values.

Compositions	Linkers	Yeast and Fungi	Tested value	References
[Ag_4_(PYDC)_2_(pm)_2] n_ H_2_PYDC	4[Ag_4_(PYDC)_2_(pm)_2_] n H_2_PYDC	*Candida albicans* *(C. albicans)*	Minimum inhibitory concentration MIC = 4 ppm	[149]
[Ag_2_(l4-PTA) (l4-MAL)] n PTA	Pure Terephthalic acid PTA	*Candida albicans* *(C. albicans)*	MIC = 30 ppm	[156]
13 Ag(l3-PTAS) (NO_3_) (H_2_O)]	PTAS	*Candida albicans* *(C. albicans)*	MIC = 30 ppm	[157]
[Ag_4_(l4-PTAS) (l5-PTAS) (SO4)_2_(H_2_O)_4_] n	PTAS	*Candida albicans* *(C. albicans)*	MIC > 60 ppm	[157]
[Cu_3_(BTC)_2] n_ (HKUST-1)	H_3_BTC	*G. candidum* *S. cerevisiae*	Growth inhibition of microorganisms GIM = 99.99 % at 48 hrs GIM = 99.99 % at 24 h	[153]
[Co(meim)_2] n_ (ZIF-67)	Hmeim CFs	C. albicans *Candida albicans*	Zone of inhibition ZOI = 17 mm (about 0.67 in)	[158]
[Zn(meim)_2_] n (ZIF-8)	Hmeim	*Candida albicans* *(C. albicans)*	Zone of inhibition = 16 mm	[158]
Ni(hydeten)_2_Au_2_(C)_4_	hydeten	F. oxysporum R. solani A. solani	GIM = 51.3 % GIM = 81.9 % GIM = 48.0 %	[159]
Cu (hydeten)_2_Au2(CN)_4] n_	hydeten	R. solani A. solani F. oxysporum	GIM = 0.0 %	[159]
[Co_4_(Glu)_4_(bpp)_2] n_	H_2_Glu bpp	A. niger C. albicans	GIM = 62 % GIM = 99.93 %	[160]
{Co_2_(Glu)_2_(bpa)} (H2O)_4] n_	H_2_Glu bpa	*A. niger* *C. albicans*	GIM = 46 % GIM = 99.98 %	[160]
[Cu_3_(BTC)_2] n_ (HKUST-1)	H_3_BTC	*F. oxysporum* *niger* *C. albicans* *A. oryzae*	GIM = 30 % No growth inhibition GIM = 99.99 % GIM = 30 %	[161]

Notes: ZOI = zone of inhibition, GIM = Growth inhibition of microorganisms, MIC = minimum inhibitory concentration.

## 6 Antiviral activities of MOFs

The ongoing COVID-19 pandemic, alongside persistent threats from established viruses like Ebola, HIV, and hepatitis B and C, underscores the critical need for robust public health interventions. The growing field of MOFs offers exciting possibilities for virus detection, a critical step in combating various diseases. These novel materials hold the potential to revolutionize diagnostic techniques and improve disease management [[32], [162]].

Horcajada et al. [163] stated that the various types of virions may be encapsulated by MOFs in their cavities and the range of their size is from tens to a few hundred nanometers, stopping their spreading and replication. Few studies have been done regarding MOFs as antiviral agents. The most recent and relevant studies among them deal with several non-toxic iron(III) based MOFs (Fe (III) MOFs), basis on different carboxylate linker such as [[Fe_3_O(OH)(H_2_O)_2_(BDCNH2)_3_]_n_, (NH_2__−_MIL-101(Fe)), [FeO(OH)(H_2_O)_2_(MCN)_3_]_n_), [Fe_3_O(OH)(H2O)2(BDC)_3_]_n_, (MIL-101(Fe)), [Fe_3_O(OH) (H_2_O)_2_(BTC)_2_)]_n_, (Fe-MIL-100) and [FeO(OH)(H_2_O)_2_(FMR)_3_]_n_. All of them are synthesized in the nanoparticle form, characterized by different degradability related and biocompatibility, and examined as antiviral drug carriers such as Cidofovir (CDV) and azidothymidine triphosphate (AZT-TP).

According to [164], Azidothymidine (AZT) is considered a prodrug and called zidovudine. The intracellular kinases metabolize this drug and convert it into the active triphosphate derivative where synthesis of the proviral DNA and retro transcription is inhibited protecting cells from HIV infections.

**Scheme 1:**
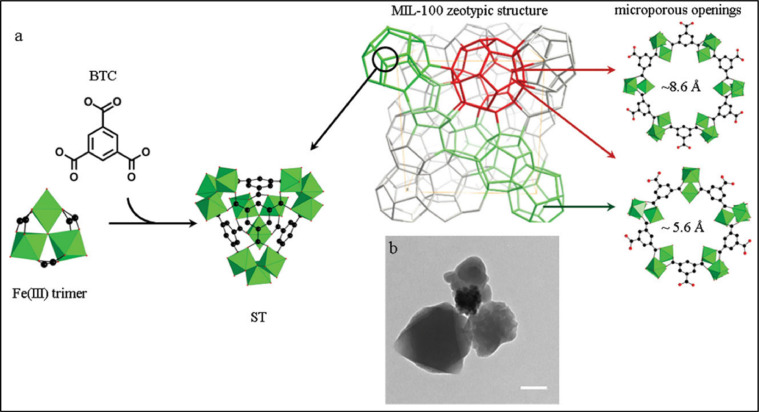
(a) Microwave-assisted hydrothermal synthesis of MIL-100′s structure and (b) MIL-100 nano MOFs observed by TEM (100 nm).

Another study was done by Agostoni et al. [165] about a MOF-based iron (Fe-MIL-100) (Fe-MOF 112), which was used in the form of nanoparticles. It was assessed additionally for azidothymidine triphosphate (AZT-TP) as molecular sponges. About 24 wt% of AZT-TP is absorbed quickly by MOF112 nanoparticles with entrapment performance and the MOF's structure does not change. Furthermore, a probable coordination seems to be proposed by the structure of the nano MOFs 12 to unsaturated Fe (II) sites after the interface with Azidothymidine (AZT) and Azidothymidine triphosphate (AZT-TP). Triphosphorylated AZT is competently penetrated and released into a targeted cell of major Human immunodeficiency virus (HIV) by the loaded nanoparticles, which protect against HIV infection. The building blocks of MIL-100 nanoMOFs are hybrid super-tetrahedra (ST), which are produced when oxo-centered trimers of iron (III) octahedra and trimesic acids spontaneously coordinate (Scheme 1).

Furthermore, [166] completed another study on the nanoparticles based on Fe (II) MOFs 12 as an antiviral agent where they investigated the encapsulation process and AZT-TP releasing as well as lamivudine triphosphate (3TC-TP) was also included. The nanoMOFs loaded with drugs were stored for two months kept freeze drying and maintained similar physicochemical features. The monocyte-derived macrophages were used to check their activity against viruses, and they were infected with HIV.

**Fig. 11:**
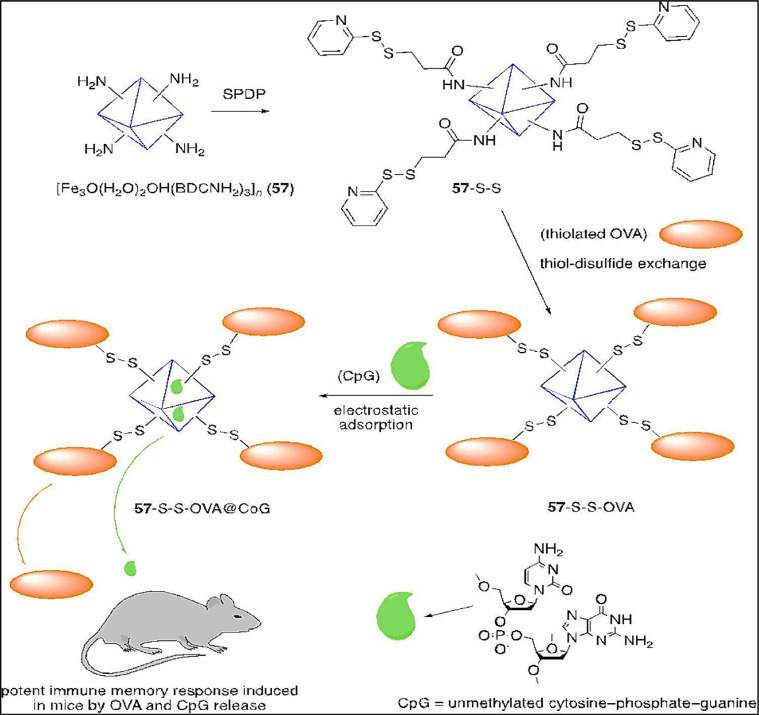
Delivery and transport system synthesis CoG@57-S-S-OVA [167].

Yang et al. [167] investigated the use of nanoparticle MOFs for controlled encapsulation and release of subunit vaccines. This approach aimed to stimulate cytotoxic T lymphocyte (CTL) production, thereby enhancing the cellular immune response. The unmethylated cytosine phosphate guanine (CpG) oligonucleotide along with the delivery of the antigen model (OVA) ovalbumin was transported and delivered by using nanoparticles of MOF [Fe_3_OH(H_2_O)_2_O(BDCNH_2_)_3]n_ and (NH_2__−_MIL-101(Fe)) as carrier agents. The S-S disulfide bonds were used for the conjugation of ovalbumin to the NPs surface of MOFs 57 by (SPDP) N-succinimidyl 3-(2-pyridyldithio)-propionate reaction whereas electrostatic adsorption was used to encapsulate the electronegative CpG in the pores of MOF 57 as shown in Fig. 11.

Another attempt was made by Zhang et al. [8]. The study created a co-delivery system for CpG and OVA involving Zn (II) MOFs 38 and (ZIF-8) utilization as nanoparticles. Where OVA was inserted in the MOF's 38 pores whereas CpG was attached by electrostatic adsorption. The effective release of CpG and protein antigens was allowed by the pH-induced decomposition. A vaccine's ability to stimulate strong cellular and humoral immunity is completed by both *in vivo* and *in vitro* tests Fig. 11 illustrates the dominant mechanisms for MOFs such as diffusion, van der Waals forces, electrostatic attraction, coordination, ion exchange, π–π interaction, and acid–base interaction.

The long-time action of composite virus-MOF38 was examined by Luzuriaga et al. [168]. The examination was done in an animal model to find out the reliability of the virus that is wrapped, its immunogenicity, and biosafety. The study of tissues was done and there was no injury recorded in tissues, skin, or any other organs in mice Jaros et al. [150] showed an example of MOFs examined as an agent used against viruses. Silver MOFs (Ag (I)-MOFs) [Ag_4_ (µ-PTA)_2_(µ_4_-PMA) (H_2_O)_8]n_ (H_4_PMA = pyromellitic acid), exposed notable action against (HAdV-36) human adenovirus. Table 5 summarizes the discussion and different types of MOFs; showing antiviral activities against different viruses with their compositions, linkers, antiviral agents, viruses that are targeted, and values of tests performed.

**Table 5:** MOFs compositions, linkers, antiviral agents, encapsulated and targeted viruses.

**Compositions**	**Linkers**	**Antiviral agents**	**Targeted Virus**	**Values of Performed** **Tests**	**References**
[Ag_4_(l-PTA)_2_(l3-PTA)_2_(l4-PMA) (H2O)_8] n_	PTA H_4_PMA	CpG and OVA	(HAdV-36) Human adenovirus 36	Virus replication inhibition VRI > 99 %	[150]
[Zn(meim)_2] n_ and (ZIF-8)	Hmeim	CpG and OVA	Activity to stimulate immunity	TNF-*a* > 6000 pg/mL IFN-c 120 pg/mL	[8]
[Zn(meim)_2] n_ and (ZIF-8)	Hmeim	CpG and OVA	(TMV) Tobacco mosaic virus	TNF-*a* > 6000 pg/mL IFN-c 120 pg/mL	[168]
(MIL-101(Fe)) [Fe_3_O(OH)(H_2_O)_2_(BDC)_3] n_	H_2_BDC	Azido thymidine triphosphate (AZT-TP) and Cidofovir (CDV)	Cytomegalovirus (CMV) retinitis Human immune virus (HIV-1-LAI)	Virus replication inhibition VRI = 90 %	[163]
[Fe_3_O(OH)(H_2_O)_2_(BDCNH_2_)_3] n_ (NH_2__−_MIL-101(Fe)	H_2_BDCNH_2_	(AZT-TP) Azido thymidine triphosphate and Cidofovir (CDV)	Cytomegalovirus (CMV) retinitis Human immune virus (HIV-1-LAI)	Virus replication inhibition VRI = 90 %	[163]
[Fe_3_O(OH)(H_2_O)_2_(BDCNH_2_)_3] n_ (NH_2__−_MIL-101(Fe)	H_2_BDCNH_2_	SPDP and CpG	(CTL) cytotoxic T lymphocyte	Virus replication inhibition VRI = 67 %	[167]
[Fe_3_O(OH)(H_2_O)_2_(BTC)_2_)] n (Fe-MIL-100)	H_3_BTC	Azido thymidine triphosphate AZT-TP	Human immune Virus- Long-acting injectable HIV-LAI	Virus replication inhibition VRI = 90 %	[165]

## 7 Antiparasitic activities of MOFs

The discussion explored the diverse applications of MOFs and their derivatives, highlighting their effectiveness against various pathogens like bacteria, fungi, and viruses. The conversation emphasized the vast potential of these materials with different linker and compositional variations in combating various parasitic threats. According to Khaligh et al. [169]; Dubey 2021; Mapossa et al. [170] both endoparasites and ectoparasites cause severe infections and diseases due to which treatment and prevention of these parasites are growing and showing interest for their development Tabrizi et al. [171] reviewed a study that was recently published. In the study, the MOFs have been used as a vector for antiparasitic agents. In one of the studies, it is mentioned that nickel (II) MOF with 2-methylimidazole (Ni (II) MOF [Ni(meim)_2]n_) has been prepared. The average size of the particle was recorded at about 761 nm. Which showed activities against the larva of the Aedes aegypti mosquito. It is noted that small-sized particles of MOFs can penetrate cells of the midgut epithelium, reducing the survival and growth rate of the larva.

**Fig. 12:**
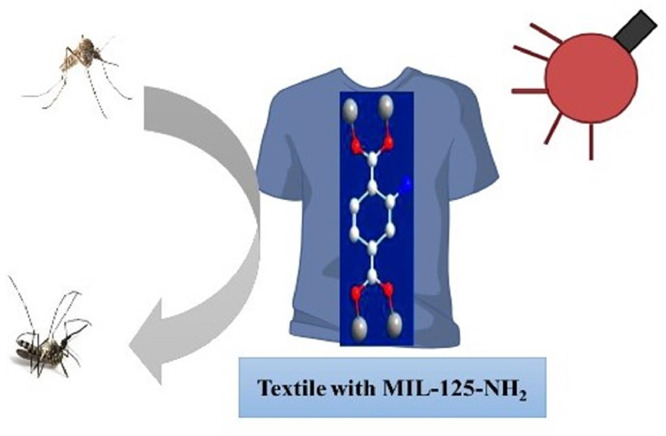
Activities of titanium organic framework (Ti-MOFs) supported on fabrics against parasites, (modified from [172]).

The apoptosis was provoked by nickel ions where cell cycle was induced, and reduction of growth rate led to the death of mosquito larva. It is found that a low dose of MOF61 is non-toxic whereas a high dose of MOF is found lethal with LC_50_ the value is found MOF138. 33 ± 3.7 µg/ml and all the processes were done naturally (*in vivo*) by using an animal model system namely Artemia salina. Another attempt was made by Abdelhameed et al. [172] where the (Titanium based MOF) Ti-MOF [Ti_8_O_8_(OH)_4_(BDCNH_2_)_6]n_ (MIL-125) was used in changed absorptions for the decoration of natural viscose, linen, and fibers cotton as shown in Fig. 12.

In earlier studies, the coating of MOF 14 crystals, and 3-glycidyl loxypropyltrimethoxysilane were used to modify the fabrics. The ready mixtures were used against the Culex pipiens, common house mosquito (*C. pipiens* Mosquitoes), and reported very effective death of mosquitos without using any insecticides. The mosquito-killing mechanism was planned as a daylight result on MOFs 14, which is formed by dimethylol dihydroxy ethylene photodegradation urea where mosquitoes are attracted to the fabrics. The fabric also showed resistance to washing enduring beyond five washing cycles as compared to normal fabric.

Moving to the next nanoparticle example of Zn (II) MOF [Zn (OAc)_2_(bipy)] n, used as cargo for an antiphrastic agent. This antiparasitic agent was prepared with sizes ranging from 28 nm - 80 nm. Also loaded mechanochemically with ergosterol peroxide (EP) and used to apply *in vitro* trypanocidal antiparasitic activities [173]. Different MOFs and MOFs-based materials show antiparasitic activities as mentioned in Table 6.

**Table 6:** MOFs with antiparasitic agents, compositions, and linkers.

**Compositions**	**Linkers**	**Antiparasitic agents**	**Parasitic**	**References**
Fe_3_O(OH)(H_2_O)_2_(BDC)_3] n_ (MIL-101(Fe))	H_2_BDC	Dinotefuran carboxymethyl chitosan (DNF CMCS) matrix	(Fam. Fulgoroidea) Planthopper	[174]
[Fe_3_O(OH)(H_2_O)_2_(BDCNH_2_)_3] n_ (NH_2__−_MIL-101(Fe))	H_2_BDCNH_2_	Curcumin curc	Toxoplasma gondii	[175]
[Zn (OAc)2(bipy)] n	bipy	Ergosterol peroxide EP	Trypanosoma cruzi	[176]
[Zn_8_O(Ad)_4_(BPDC)_6_(NH2(CH_3_)_2_)_2] n_	HAd H_2_BPDC	Art Fe_3_O_4_ matrix	Leishmania major	[177]
[Zr6(O)4(OH)4(BDCNH2)_6_] n	H2BDCNH_2_	curc	Toxoplasma gondii	[175]
[Ni(meim)_2]n_	Hmeim	–	mosquito larvae Aedes aegypti	[178]
Fe_3_O(H_2_O)_2_OH(BTC)_2] n_ (Fe-MIL-100)	H3BTC-FIT H3BTC	Azox	*P. infestans* *F. graminearum*	[179]
(Ti_8_O_8_(OH)_4_(BDCNH_2_)_6_]n (MIL-125)	H2BDCNH2		*C. pipiens* Mosquitoes	[155]
[Zn (OAc)_2_(bipy)]_n_	[bipy EP	EP Ergosterol peroxide	Trypanosoma cruzi	Ramos et al.*,* 2012
[Zn_8_O(Ad)_4_(BPDC)_6_(NH_2_(CH_3_)_2_)_2_] n (UiO-66-NH_2_)	HAd H2BPDC	Glt Fe3O_4_ matrix	Leishmania major	[180]

## 8 Use of MOFs for food preservation

Silver-based MOFs are used in research by Lu et al. [181] to extend the shelf life and preserve fruits, and because of their broad-spectrum nature, they make an ideal antimicrobial agent. However, according to Zhang et al. [43], most research on Ag-MOFs focuses only on antimicrobials, including all microorganisms such as bacteria and fungi, rather than antibacterial only.

However, the water instability and powder form of MOFs also affect the action of MOFs against different microbes. To avoid the powder and aqueous instability problems, two methods were used by André et al. in 2019. One method was stabilizing the dispersed form of MOFs by adding surfactants. Two surfactant agents i-e (octadecylphosphonic acid and behenic acid) were used for the reduction of particle aggregation and the improvement of stability. Another method was used for immobilizing MOF on a solid substrate.

**Fig. 13:**
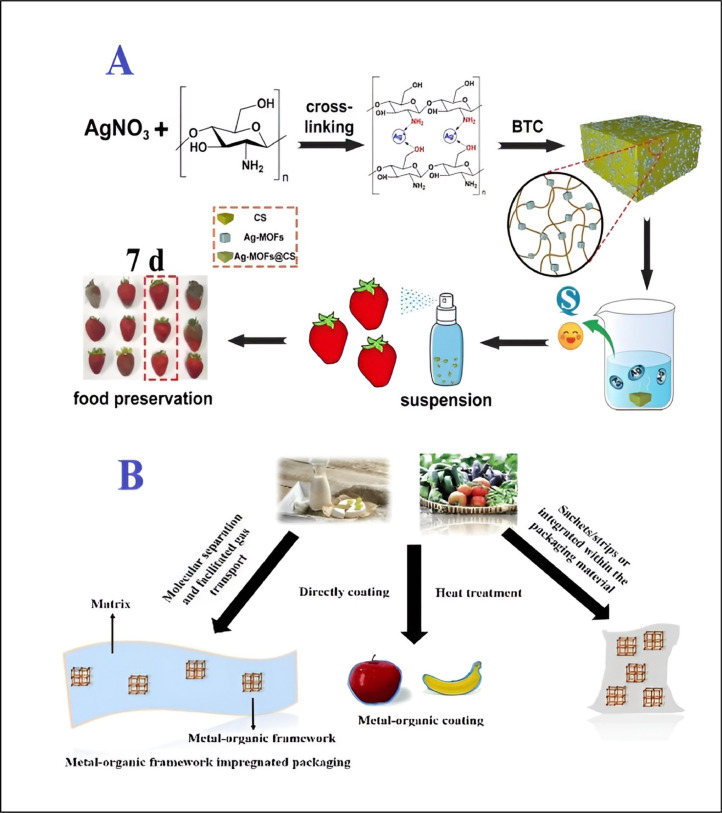
(A). exposes the (Ag-MOFs@CS) silver-based MOF synthesis and the process of spraying for fruit safety [12] and (B). MOFs incorporation in packaging material for food quality preservation: Sachets containing a MOF, MOF-impregnated packaging, coating with MOFs [184].

In the vision of the problems mentioned above, the (Ag-MOFs) silver-based MOFs with chitosan (CS) which is a natural polymer is the best technique to sort out the problems of Ag-MOFs with bad aqueous stability for the use against bacteria that cause infections and food spoilage. On the other hand, chitosan has other beneficial applications such as they are easily available, economically good, non-toxic, and playing roles in biodegradation [[12], [182], [183]] explained the method for the Ag-based MOFs@CS preparation with the stability of water and with their best antimicrobial activity. They explained the silver ions' interaction with a group of amino acids along with hydroxyl groups in chitosan (CS) to achieve and enhance the stability of silver-based MOF (Ag-MOFs) for the preservation of fruits from different bacterial infections, as exposed in Fig. 13A.

For food packaging, MOFs and MOFs derived materials can be incorporated with three different methods. Sachet with MOFs saturated in the packaging matrix and incorporated in a coating as shown in Fig. 13(B). MOFs incorporation in food packaging materials can be used for the following purposes such as moisture absorption, O_2_ treatment, and gas separation, or can be used as an antimicrobial agent, etc. The utility of polymer membranes in packaging substrate is constantly limited by requiring a balance among the selectivity and permeability, whereas the exclusive properties of absorbance selectivity (facilitated gas transport and molecular separation) are combined by hybrid matrix membranes (HMM) and the mechanical stability and processability of polymers Datta et al. [185]. According to Rodenas et al. [186], Cu-BDC nanosheets were reported as the 1st materials, which were saturated with a polymer matrix in the procedure of preparing mixed matrix membranes aimed at carbon dioxide separation from CO_2_/CH_4_ mixtures.

Chopra et al. [187] reported that molecules on the surface including activated carbon or potassium permanganate always absorb ethylene. MOF materials are used in the packaging/sachet strips. The bananas ripening induced by ethylene, where ethylene is bound to C300, demonstrates the possibility of releasing volatile compounds from MOFs for applications in practical.

In the case of coatings, the necessary MOF coating materials may often be prepared by directly coating them on suitable substrates after a straightforward heat treatment Yang et al. [188] selected gelatin hydrogel coated with UiO-66NO_2_ to prepare robust MOF film by process of straightforward treatment of heat. The composite gelatin/ UiO-66-NO_2_ demonstrated increased efficiency by removing lead (II) from apple juices etc. This coated film exhibits negligible impact on the apple juice quality as compared to an uncoated film.

## 9 Mechanism of antimicrobial activities of (MOFs) and MOFs-Derived materials

After an overall discussion of MOFs and MOFs based on their antimicrobial activities including bacteria, fungi, viruses, and parasites. There must be a methodology or mechanism of action such as how these MOFs work against the targeted microorganisms Willdigg et al. [189] and Mba et al. [190] stated that the type of bacterial cell wall, composition, and structures deeply influence the antimicrobial activity of any agent used against them.

Karimi et al. [191] proposed that each single MOF has its specific action against specific microbes such as viruses, bacteria, and fungi etc., some of them are identified in the different groups of MOFs analyzed with their specific structure and compositions and especially linked to the cell wall of bacteria either gram-positive or gram-negative bacteria. Depending on the difference between the gram-negative and gram-positive is the peptidoglycan thickness. The cell wall of gram-positive bacteria is thick whereas the gram-negative bacteria possess only one outer membrane. Peptidoglycan with thick layers prevents the metal ions or nanoparticles from entering the cells released by MOFs. Generally, the cell wall of gram-negative bacteria is observed with a larger zone of inhibition and lower MIC values.

**Fig. 14:**
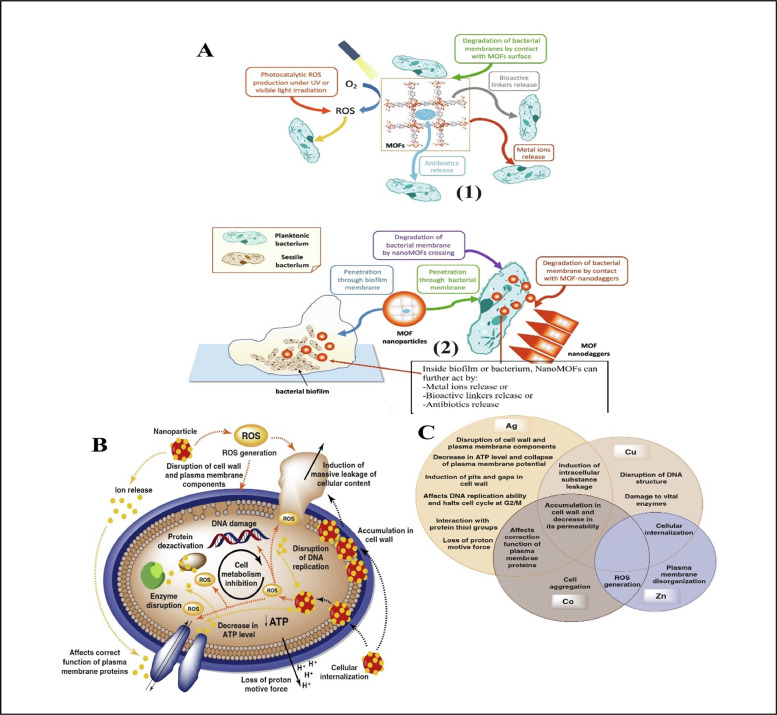
A: (1) Different MOFs exert the possible mechanisms of antimicrobial as nanoparticles or microcrystalline powders in compound materials such as Top of Form over metal ions release or linker with basic antimicrobial action over progressive breakdown of the crystalline frame of the MOF, control release of Microbial agents helps to encapsulated in the permeable MOFs structure in control way, A (2) Shows that how NPs of MOFs having proper size manage to enter into the bacterial cell wall by changing membrane and causes degradation, enter into the walls and apply antimicrobial action against the bacteria and all this is possible through the releasing of metal ions as well as linkers and antimicrobial molecules are encapsulated in nanoparticles MOFs, (B) Nanostructured systems with metals and their antimicrobial activities and antibacterial mechanism of different metals, (C) Mechanism of (Ag, Cu, Zn, and Co) which are used for metal action along with the for metal nanoparticles [37]. ROS: Reactive oxygen species.

Pettinari et al. [192] proposed a study about the Ag-MOFs and considered them more effective against gram-negative bacteria including *E. coli* and *Pseudomonas* as compared to gram-positive *Staphylococcus.* According to their study, the silver ions can penetrate the bacterial cell wall and destroy it. Antimicrobial activity is also stimulated by the morphology of the MOFs, as more Ag^+^ ions are released by MOFs with high surface area. Furthermore, MOFs with the size of nanoparticles can easily enter the cells of bacteria, decrease permeability, and cause cell lysis and bacterial death. A diagram mechanism of MOFs is presented in Fig. 14A (1 and 2).

The potential MOF mechanisms against the various bacteria are depicted in Fig. 14A. Various MOFs in the form of nanoparticles in composite materials or microcrystalline powder exert this process. This technique can be accomplished via the release of biocidal chemicals that are enclosed inside MOF's porous structure, or by the gradual disintegration of MOF's crystalline lattice, which releases metal ions or linkers. When MOF nanoparticles are the right size, they can penetrate bacterial membranes and cause degradation, which alters the membrane potential, as shown in Fig. 14A. In addition, they penetrate the biofilm walls, whereupon they initiate antibacterial activities by releasing metal ions in a sessile form, biocidal compounds, or a linker that is encased in the release of MOF's nanoparticles Pettinari et al. [192]. The same mechanism to interrupt the membrane of bacteria was confirmed with the prepared MOFs in nanodaggers form Pettinari et al. [192]. MOFs can control antibiotic resistance with different physiochemical methods and produce effective antibacterial activity depending on several factors such as metal ions and ligands, physical contact, photothermal effect, and oxidative stress. Antibacterial activity can also be enhanced with a synergetic effect with several mechanisms that are explored and used. Fatty acids and proteins in membranes of bacterial cells were susceptible to transmembrane potential or oxidation might be different due to insoluble water strong Cu-MOFs crystals along with metal active sites attached to the surface of bacteria and bacterial cell death/inactivation occurs due to this attachment.

Four 3D Cu-MOFs intended as [Cu_2_ (Glu)_2_ (μ-L)] ⋅x (H_2_O)] were synthesized by Jo [143] and their antibacterial activity was investigated against different strains of bacteria. Five strains were tested including *S. aureus, K. Pneumonia, Methicillin-resistant S.aureus (MRSA), E. coli,* and *P. aeruginosa* where it is noted that their growth was completely suppressed with bacterial killing efficacy of 99.9 % at (MBC) minimum bactericidal concentration of 20 μg/mL. The higher action against bacteria primarily results from the surface metal site of Cu-MOFs, which is active rather than leaching Cu^2+^ ions.

MOF serves as a delivery system for drugs. Typically, medications are added to MOFs in a post-synthesis manner. MOFs are filtered out in a pre-synthetic technique, after their stirring in medicinal solutions. The main advantage of this process is that the dosage of the medication may be adjusted by varying the stirring and concentration times. However, some MOFs can be disintegrated during the post-synthetic method Velásquez-Hernández et al. [133]. MOFs can be loaded in concerned drugs in a controlled environment for the better result. Antimicrobials can be used as MOF components like ion or rental. For example, in the year 2019, nalidixic acid, which is 1st generation quinolone and a broad-spectrum antibiotic used as a linker in manganese (Mn) and magnesium (Mg) base MOFs André et al. [140].

In a different investigation on drug-loaded MOFs, Bhardwaj et al. [148] loaded three Zn-MOFs—Isoreticular metal-organic framework-3 (IR-MOF-3), zinc (II) benzene dicarboxylate (Zn-BTC), and MOF-5—with broad-spectrum antibacterial antibiotics, such as kanamycin and ampicillin. In 2018, Soltani and associates carried out an additional investigation and noted that gentamicin loaded with zeolitic imidazolate framework-8 (ZIF-8)—a class of metal-organic frameworks (MOFs)—displays a positive and strong activity against bacteria, with 12 mm (about 0.47 in) and 14 mm (about 0.55 in) of inhibition zone recorded against *E. coli* and *S. aureus*. The medications that were given and the drugs that were loaded with MOFs were discussed above. We now shift our attention to another MOF antibacterial mechanism.

In this mechanism of action against different microbes, Photosensitizer compounds are used by MOFs. Photoelectric molecules are absorbed by these molecules and transfer to their surrounding molecules. The photosensitizer molecules help in the energy transfer from the ground state having triplet oxygen (^3^O_2_) to single-state oxygen (^1^O_2_), which causes oxygen damage to living cells Lismont et al. [193]. According to [194], these photosensitizer molecules are used as photodynamic therapy on a large scale to kill different microbes. Furthermore, there are many ways to improve MOF's photoactivity for various applications against bacteria, such as structure changes as well as with the noble metal nanoparticles combination. An example is given here. Mao et al. [195] doped Ag nanoparticles based on zirconium-porphyritic MOFs simple as ZPM.

Following a brief overview of photosensitizers, this study delves into the application of MOFs as chelating agents. By chelating metal ions, MOFs reduce their positive charge. This reduction in positive charge facilitates the binding of the MOF-photosensitizer complex to the negatively charged cell wall, enhancing its effectiveness Patel et al. [196]. Anticancer drugs have been designed using a chelating process, which includes cisplatin a platinum-based drug that forms chelate with DNA or in the form of a detox agent, which helps in the elimination of a huge quantity of toxic ingredients in gastrointestinal. Many Cu-MOFs are used against bacterial infections [143]. The action of MOFs against the bacteria is related to the bacterial cell damage physically not to metabolic processes such as with antibiotics, which occur through different mechanisms as presented in Fig. 14 (B and C). The mechanism of antimicrobial nanostructured systems consisting of metals is labeled here. Metal oxide nanoparticles have some antimicrobial action. As previously discussed about the Ag-MOFs. The antimicrobial mechanism depends on the Ag^+^ release, which is followed by the permeability of the membrane, efflux of phosphate, cell de-energization, DNA disruption replication, and cellular contents leakage Li et al. [197].

As per the findings of Sondi and Salopek Sondi et al. [198] and Ruparelia et al. [199], once Ag-NPs pass through the cell, the DNA becomes a condensed state that inhibits replication and ends the cell cycle. According to Halevas et al. [200], Zn (II) based on Schiff exhibited antibacterial activity against both gram-positive and gram-negative microorganisms Yamamoto et al. [201] used two distinct species *E. coli* and *S. aureus* to investigate the antibacterial properties of various (ZnO) zinc oxide nanoparticles of varying sizes. The scientists observed that the actions against bacteria increased as the size of ZnO particles shrank. This becomes possible with the efficiency of hydrogen peroxide (H_2_0_2)_ on the ZnO surface. The H_2_O_2_ causes damage to the cell walls and their structure by easily penetrating bacterial cells Brayner et al. [202] stated that the cell is damaged by the interaction of the ion released of Zn^2+^ and causes interaction with contents inside the cell Raffi et al. [203] recognized the Copper nanoparticles and their best antibacterial activity. Cu^2+^ ions are released by copper NPs to target bacteria. The copper spices which are soluble show actions against microbes, and they depend on the speciation of soluble species. This mechanism results in cell lysis and cell ruptures where cellular materials are released by using different gram-negative and gram-positive bacteria Gunawan et al. [204].

Regarding the cobalt nanoparticles and their action against different microbes, Khan et al. [205] showed that negligible antibacterial activity was noticed in Co_3_O_4__—_NPs (MIC > 10,000 ppm). Whereas Gouda et al. [206] indicated that after a short time of 1 min contact, the CoO NPs spread over cotton fabrics were toxic for *E. coli* and *S. aureus.* The above oxides are discussed with their mechanism antimicrobial actions as shown in Fig. 14.

After antibacterial activities and other medical advantages, MOFs has also many other environmental benefits. It is understood that industries release many toxic chemicals and gases. This affects the living organisms and the green environment. Commonly liquid alkanolamine is most often used for the adsorption of CO_2_, which is not enough to meet the requirement for green environment development. According to Xu et al. [207], an American company for the 1st time developed Amino-beta-cyclodextrin (NH_2_-β-CD-MOF). It was synthesized by using amino-functionalizing β-CD, which was cost-effective, easily available, and in biocompatible form. It is noted that the adsorption quality of NH2-β-CD-MOFs for CO_2_/N_2_ was better proof. X-ray photoelectron spectroscopy (XPS) and Fourier transform infrared spectroscopy (FT-IR) were used to determine the mechanism of adsorption for N_2_ and CO_2_. The highest reported CO_2_ performance for NH_2_-β-CD-MOFs was 12.3 cm^3^, a ten-fold improvement.

MOFs are employed for gas separation in addition to several harmful gas adsorption techniques. According to Li et al. [103] and Shi et al. [208], MOFs have a huge surface area, are stable at high temperatures, are modular, and have exceptionally high porosity. It is the best option since it can separate gases. MOF membranes are classified into two groups: pure MOF membranes and mixed matrix membranes, for gas separation. Pure MOF membranes are stable on chemical and temperature levels and are good physically and chemically for different environmental/industrial applications but with limited use in different applications due to their high cost, complex process of preparation, and difficulties. On the alternative, a mixed matrix membrane is an easily available, low-cost membrane, ideal process, and possesses constant pore size of inorganic materials. Miller et al. [209] stated that MOFs have been designed for different gas storage in initial phases. More applications for gas storage have been reported, such as carbon dioxide, hydrogen, and oxygen storage. MOFs are also used for sensing purposes to detect biomolecules and inorganic.

## 10 Toxic effect of MOFs

While the discussion highlighted the numerous advantages and promising applications of MOFs and MOF-based materials, it is important to acknowledge that they also present certain drawbacks. Porous materials should be chemically stable, water stable, and have exclusive MOFs for different factors in practical applications Haldar et al. [210]. Metal-organic coordination bonds are attacked by water molecules where crystal phases are changed, ligand metal bonds are broken and finally, the structure of MOFs are collapsed [[52], [211], [212]]. According to Kumar et al. [213], there are still issues with MOF synthesis that need to be fully addressed before they can be resolved on an industrial scale. These issues include toxicity, recycling, reuse, and degradation processes.

The toxicity associated with MOFs is poorly understood and it needs serious attention and control for better environment and human health. The reason for MOF toxicity is the occurrence of functional groups and metal ions in organic ligands [[214], [215], [216]]. Another disadvantage of MOFs is the decrease of the crystal to the nanoscale. As the size of the crystal increases, they become more reactive and toxic. MOFs possess small nanoscale sizes, which can enter human cells easily where they can cause different toxicity. Nanoparticles have a very invasive nature depending on their size. According to research, nanoparticles less than 35 nm in size can easily penetrate blood-brain barriers, and a size less than 40 nm can get easy access to cells/nuclei. Furthermore, nanoparticles having a size of less than 100 nm can cross the cell membrane easily [[216], [217]]. Following a thorough investigation, it is determined that MOFs have benefits and drawbacks, which are enumerated in Table 7.

**Table 7:** Toxic effects of MOFs.

**MOFs**	**Detection index/Recycle/Reuse techniques**	**Applications/Toxicity**	**References**
MIL-88A, 88B_4CH_3_, MIL-100	Solvent exchange and warm treatment	Delivery of drugs	[218]
MOF-177 compound	Exchange of solvents, thermal and vacuum treatment	Storage and separation of gases	[219]
MOF-19 compound	Treatment of vacuum and Solvent exchange,	Catalysis	[219]
Zn_4_O(ADC)4 (Et_3_N)6 (IRMOF-0)	vacuum treatment and solvent exchange	Storage of Gas	[219]
IRMOF-16	Activation by Supercritical Carbon Dioxide (SCCO_2_)	storing of Methane	[220]
Zn_2_ (DHBDC). DMF·2H_2_O, (MOF-74)	Conversation of solvent	Adsorption of gas	[221]
Co (1, 4-BDC). DMF, (MOF-71)	Conversation of solvent	Non-linear optics, Hydrogen sorption, catalysis, and magnetism	[221]
Zn_2_ (DHBDC)(DMF)_2_. (H_2_O)_2_, (MOF-74)	Conversation of solvent	Adsorption of gas	[222]
Mn_3_(BDC)_3_ (DEF)_2_, (MOF-73)	Air drying and conversation of solvent	Magnetization	[[221], [223]]
Pb(1,4-BDC) (C_2_H_5_OH) ‚ (MOF-70)	Conversation of solvent	Non-linear optics, Hydrogen adsorption, catalysis and magnetism	[[221], [224]]
ZIF-8	–	Slow release of drugs and Metal ions	[225]
UiO-66, UiO-67, Co-MOF-74 Mg-MOF-74	Embroys of Zebrafish	Barely toxicity	[226])
MG-Gd-pDBI MOF	Body serum and blood parameters	Low toxicity of blood	[227]
[Eu (BTC)] MOF	Biochemical and blood parameters (reanal and hepatic function)	No toxicity	[228]

## 11 Conclusion and future perspectives

Microbial infections pose a significant global threat, often characterized by high mortality rates and increasing concerns about antibiotic resistance. In response to this growing challenge, the past two decades have witnessed significant advancements in the development and application of Metal-Organic Frameworks (MOFs) and MOF-based materials as promising antibacterial agents.

The properties of MOFs and MOF-based materials, such as the controlled release of antimicrobial components, tunable nanomaterial sizes, varying morphology, production of reactive oxygen species, capacity to load and deliver various agents used against various microorganisms, and alternative drug delivery, all influence their efficacy. These qualities make MOFs and their derivative materials the ideal option when it comes to antibacterial agents. To direct the development of next-generation MOF-based materials, their practical usage, and applications, the antibacterial, antiviral, and antifungal properties of MOFs as well as MOF-derived materials are covered in depth. Because MOFs are tailored, they have unique benefits over other antibacterial agents and show long-lasting and focused antibacterial activity; nonetheless, there are still some obstacles to be addressed.

A recent review analyzing research up to 2023 identified several MOFs and MOF-based materials with exceptional potential as broad-spectrum antimicrobial agents. These include Cu-MOFs, ZIF-8, Cu-MOF nanoparticles, Zn-BTC, Co-SIM-1, and Ag(I)-based materials. Notably, these materials demonstrate remarkable activity against a wide range of bacteria, viruses, and fungi. Furthermore, the study explored the use of specific MOFs, such as Zn-MOFs, IR-MOF-3, Zn(II), Zn-BTC, and MOF-5, as drug carriers. These MOFs can be loaded with various pharmaceuticals or act as carriers for antibiotics, such as nalidixic acid.

This review delves into the antibacterial mechanisms, diverse synthesis methods, and characterization techniques of MOFs. It also emphasizes the broad-spectrum antimicrobial activity of both MOFs and MOF-based materials. The findings highlight MOFs as promising alternatives to traditional antibiotics due to their: Reduced potential for resistance development, physical disinfection capabilities, and drug delivery potential for targeted antimicrobial therapy. However, the study acknowledges potential drawbacks associated with certain MOFs, including toxicity concerns related to solvent exchange oxidative stress, challenges in stability, usage, and recyclability.

Despite the promising potential of MOFs against microorganisms, several aspects require further exploration to optimize their applications and design. These are (1) precise control over MOF properties including the chemical and physical characteristics of MOFs that need to be precisely controlled during design to ensure consistent and reliable performance. This is crucial for various applications, as exemplified by the need for larger MOFs in certain antibacterial contexts, and (2) scalable and sustainable synthesis, which includes developing efficient and environmentally friendly methods for large-scale MOF production is essential for clinical use. This includes minimizing energy consumption and addressing potential hazards associated with the synthesis process.

A high surface area in MOFs is vital for increasing their catalytic activity and adsorption capacity. This characteristic proves important in gas separation, storage, and heterogeneous catalysis applications. The review shed light on how understanding and raising MOF surface area contribute significantly to proceeding with their efficacy and performance in several industrial and scientific contexts. The importance of pH-stable MOFs is emphasized by their crucial role in various applications. In gas separation and storage, the occurrence of basic and acidic impurities requires robust MOFs to ensure effective performance. The pH-stable MOFs enhance their importance in different applications and research fields.

The synthesis methods of MOFs have thoroughly been described in the review where different techniques have been discussed including room temperature, hydrothermal, solvothermal, microwave heating, mechanochemistry, ultrasonic, and electrochemical. Two methods are traditionally used solvothermal and non-solvothermal. The microwave method has proven significant for reducing synthesis time. The electrochemical method used for the synthesis of HKUST-1 with distinct properties. Furthermore, mechanochemical synthesis is utilized as solvent-free for fine-crystalized products. The sonochemical method reduced synthesis time and increased production rate. Overall, the diverse methods of synthesis participated in the advancement of research on MOFs.

The study emphasizes the crucial role of characterization techniques in understanding the properties of MOFs. These techniques, including FE-SEM, HR-TEM, FTIR, and PXRD, provide valuable insights into (1) morphology and structure: FE-SEM and HR-TEM offer detailed visualization of the surface features and structural characteristics of MOFs, allowing observation of particle size and shape, (2) crystallinity and structure: PXRD helps determine the crystallinity and structural parameters of MOFs, crucial for understanding their overall organization, and (3) chemical composition: FTIR spectroscopy provides information about the molecular configuration and functional groups present within the MOFs.

As highlighted earlier, most of the reported research on MOFs against bacteria focuses on infections-related wound treatment or antibacterial coating. This is mainly confined to basic *in vivo* and *in vitro* applications. However, to demonstrate further the abilities of these MOFs against microorganisms, it is necessary to modify and optimize the MOFs further for transportation through the blood circulation and go through other biological barriers for the treatment of other different diseases such as syphilis, pneumonia, and acne. Once the relation between the chemical and physical properties of MOFs and their antimicrobial properties have been established as well as their safety profile is properly studied, MOFs could potentially emerge as therapeutic agents on a clinical basis. MOFs may be used independently on their own or supplemented with a minimal dosage of antimicrobials for combating any microbial infection. This approach has positive prospects for eliminating the persistent problem of antibiotic resistance in the future.

The study highlights the green biomaterial principles and their connection with MOF synthesis. These principles convey a commitment to environmentally conscious and sustainable practice in the synthesis of biomaterials, particularly MOFs. It highlights minimizing toxic waste, using green solvents, using renewable resources, and designing nontoxic and biodegradable materials. Overall, these principles guide the development of biomaterials with a keen awareness of ecological and health considerations.

## CRediT authorship contribution statement

**Muhammad Hubab:** Data curation, Investigation, Validation, Writing – review & editing. **Mohammad A. Al-Ghouti:** Conceptualization, Supervision, Visualization, Data curation, Investigation, Validation, Writing – review & editing.

## Declaration of competing interest

The authors declare that they have no known competing financial interests or personal relationships that could have appeared to influence the work reported in this paper.

## Data Availability

No data was used for the research described in the article.
